# Building courage, strength, and knowledge: Mindfulness training reduces psychological threat and increases engagement in college physics

**DOI:** 10.1073/pnas.2521857123

**Published:** 2026-04-06

**Authors:** Tessa M. Benson-Greenwald, Avital Pelakh, Michael J. Tumminia, Sara Jahanian, Michael S. Diamond, Eric Kuo, Melanie Good, Timothy J. Nokes-Malach, Brian M. Galla

**Affiliations:** ^a^Learning Research and Development Center, University of Pittsburgh, Pittsburgh, PA 15260; ^b^Department of Physics, University of Illinois Urbana-Champaign, Urbana, IL 61801; ^c^Department of Physics & Astronomy, University of Pittsburgh, Pittsburgh, PA 15260

**Keywords:** psychological threat, mindfulness, engagement, STEM, intervention

## Abstract

Many college students experience introductory physics as psychologically threatening. In a preregistered RCT, we applied the biopsychosocial model of challenge-threat to describe patterns of threat in introductory physics and test whether a 5-d mindfulness training could reduce threat and increase engagement among undergraduates. Course-wide surveys (N = 954) screened students for the RCT and revealed roughly half of students experience psychological threat. Students identified with systemically excluded groups were more likely to experience psychological threat while systemically advantaged students were more likely to experience psychological challenge in their introductory physics course. In the RCT (N = 149), mindfulness training reduced psychological threat and fostered greater engagement in introductory physics, and, consistent with our theory of change, mindfulness training was associated with greater physics engagement through reductions in threat. The results demonstrate that mindfulness can help students manage stress more effectively by reducing psychological threat and fostering engagement in contexts like introductory physics.

## Significance

Many college students experience introductory physics courses as psychologically threatening contexts. Yet, succeeding in these high-stakes gateway courses is necessary to declare majors in engineering and physical sciences. We tested whether mindfulness, which centers attention to subjective experiences, can help provide students with tools to navigate their physics-related emotions. Course-wide survey data revealed that over half of students felt psychologically threatened in their physics course. In a preregistered RCT, a 5-d mindfulness training reduced students’ feelings of threat and increased their course engagement. This work suggests that mindfulness can support learning and persistence in Science, Technology, Engineering, and Math (STEM) courses by helping students build resilience in how they interpret and respond to stress.

*Always remember, you are braver than you believe, stronger than you seem, and smarter than you think.*
- Christopher Robin to Winnie the Pooh (1)

Large-enrollment introductory physics courses have an intimidating reputation for many undergraduate students (2). As gateways to engineering and physical science majors, these courses carry high stakes for students’ academic trajectories. Such pressures can create a “threat in the air” leading many students to experience worries and self-doubts unique to introductory physics (2, 3). For some, these doubts can contribute to disengagement – providing short-term relief at the expense of longer-term success. In the face of high demands, helping students remember they have the courage, strength, and knowledge to achieve their goals may empower them to remain engaged with their physics coursework.

Mindfulness training may provide such a pathway, giving students tools to navigate their physics demands and promote greater physics engagement. Mindfulness training, which teaches practices for attending to present-moment subjective experiences (4), promotes resilient responses to stress in clinical settings (5). However, fewer studies have examined its impact among stressed college students in STEM educational contexts (e.g., refs. 6–8). The current research tests whether mindfulness can alter how college students evaluate their physics experiences using the biopsychosocial model of challenge and threat [hereafter challenge-threat model; (9–11)]. In a preregistered, confirmatory randomized control trial (RCT), we hypothesized that mindfulness training would reduce college students’ psychological threat and, in turn, boost their engagement in introductory physics.

## Psychological Threat in Introductory STEM Courses

The challenge-threat model provides a framework for understanding how physics students’ cognitive appraisals shape stress responses (9–11). According to the model, people appraise situational demands (e.g., uncertainty, difficulty, effort) relative to their coping resources (e.g., familiarity, knowledge, support) which produces stress states. Psychological threat emerges when demands exceed resources. Conversely, psychological challenge occurs when resources meet or exceed demands. Threat is associated with greater avoidance, negative affect, and lower self-esteem whereas challenge is associated with greater approach, positive affect, and self-esteem (10, 12).

Both person-level and context-level factors can influence situational appraisals and the resulting stress responses. Environmental cues in introductory physics [e.g., high-pressure timed tests; (13–15)] can predispose many students to threat. Students often perceive these courses as competitive learning environments (16) where success requires innate abilities (17, 18) or brilliance (19–21). When students assume these beliefs are widely shared among instructors and peers, they may interpret their own difficulties as signals they cannot succeed or belong in physics (e.g., refs. 16, 18, 22, and 23). Students identified with systemically excluded groups (e.g., students of color) may be particularly likely to experience physics threat given group-based, STEM-related stereotypes (3, 15, 23–25). These beliefs and the “pressurized” environment can contribute to many students experiencing threat in physics. Interventions targeting student beliefs can create pathways for altering stress appraisals and responses (12). While other interventions have helped students reframe their stress physiology (10, 26) or affirm core values (27, 28), our intervention aimed to help students navigate difficult emotions experienced during learning setbacks by allowing them to reframe these experiences as normal and temporary parts of the learning process.

Reducing threat may help facilitate student engagement. Engagement is a multidimensional construct encompassing active cognitive, behavioral, and emotional involvement in learning (29–31). Engagement is critical for student achievement (23, 29). For example, self-efficacy positively predicts subsequent learning (32) and interventions that enhance student belonging can improve student GPA (33). We investigated whether mindfulness training can foster engagement along multiple dimensions, using theory to select and hypothesize which engagement constructs might benefit from mindfulness training. Unlike other educational interventions targeting one or two aspects of engagement, we take a more holistic, multifaceted approach, testing whether mindfulness may be a “common-ground strategy” for promoting engagement (34).

## Reappraising Physics Stress Using Mindfulness

Why might mindfulness reduce students’ psychological threat? Mindfulness training involves developing self-regulated attention and awareness toward subjective experiences (4). It fosters observing and investigating thoughts and emotions with openness and curiosity, witnessing them as ephemeral and contextual. By allowing observation and connection with difficult emotions and thoughts (e.g., refs. 35 and 36), mindfulness may enable the psychological distance needed to help students normalize and not identify with their difficult physics experiences, instead interpreting them as temporary and situational.

Optimizing college students’ responses to stress might help improve their physics experiences. In clinical settings, mindfulness is often used to treat excessive stress that may exacerbate diagnoses such as chronic pain and depression (see ref. 5 for review). However, in educational settings, stress can be adaptive, creating opportunities for growth (11). Here, mindfulness might focus on altering the type of stress response – moving from threat to challenge. In performance contexts, mindfulness trainings foster active coping (37) and reduce distracting thoughts (38). Thus, mindfulness may usher students toward harnessing stress as a challenge to overcome and promote their engagement.

Using the challenge-threat model, we considered whether mindfulness affords students opportunities to experience their physics courses differently. We expected that encouraging college students to recognize and understand their negative thoughts and emotions about physics as situational, transient, and common would foster new interpretations of physics demands and resources. The mental space and alternative processing strategies afforded by mindfulness (e.g., refs. 35 and 36) may also promote engagement. Thus, mindfulness training may reduce physics students’ psychological threat and increase engagement.

## Threat-Based Participant Sampling: Reflecting Individual and Group-Based Variation

Though introductory physics may elicit psychological threat among college students (17, 18, 20, 22), not everyone will feel threatened. Consequently, our experiment specifically included only psychologically threatened physics students for two reasons. First, mindfulness interventions have a greater impact among people with elevated stress (5). We anticipated that threatened students would be more motivated to engage with the sustained effort and practice mindfulness requires (39). Second, targeting threat may foster equity and inclusion in STEM among students identified with systemically excluded groups. Rather than targeting membership in a particular identity group, we intervened on the psychological process of threat. This decision recognizes that threat experiences vary individually—not all students from excluded groups experience threat, just as not all students from advantaged groups experience challenge. Recruiting based on psychological threat thus accommodates both individual variability and context-driven, group-based differences. As a result, this sampling procedure resulted in recruiting more students from systemically excluded groups, given anticipated between-group variation, but did not essentialize individuals by this group membership (40).

## Current Research

The current preregistered RCT tested the impact of mindfulness training on psychological threat and physics engagement. Based on the results of a pilot study (*SI Appendix*), we hypothesized mindfulness training (vs. control) would reduce threat and promote engagement. Further, we expected intervention-driven increases in engagement to occur, in part, through reduced threat. We also provide evidence for presence of a threat in the air (3) in introductory physics. Using screening data, we describe the challenge-threat distribution, exploring the prevalence and variation of threat within and across systemically excluded or advantaged groups.

Introductory physics students who screened as psychologically threatened (i.e., reported physics demands exceeding available coping resources) were enrolled in a 5-d study (Monday through Friday) and randomly assigned to either mindfulness training or a no-training, audiobook control. Mindfulness training participants received five 20-min audio lessons, delivered on Days 1 (Monday) through 5 (Friday). Audiobook control participants listened to 20-min short stories on Days 1 and 5. During Days 2 (Tuesday) to 4 (Thursday), all participants completed assessments of momentary psychological threat twice daily. Surveys at baseline (Day 1), posttest (Day 5), and 2-wk and 3-mo follow-up assessed the long-term impact of mindfulness training on threat and physics engagement.

## Analytic Plan

First, we describe the challenge-threat distribution in introductory physics courses. These descriptive and exploratory analyses use data from our screening survey (N = 954; see *SI Appendix*, Table S1 for demographics) that was administered broadly to students enrolled in 11 calculus-based introductory physics courses to determine eligibility for the main RCT. Because a large portion of our population of interest took this survey, these data present a unique opportunity to understand the prevalence of psychological threat among introductory physics students. In these exploratory analyses, we also investigate whether differences in threat emerged among students identified with systemically excluded (students of color, white women/nonbinary students) or advantaged (white men) groups (*SI Appendix*).

Second, we investigate the effects of mindfulness training among students experiencing psychological threat who enrolled in our RCT (N = 149). Two separate preregistrations contain the analytic plans for testing our core hypotheses regarding momentary and longitudinal psychological threat (https://osf.io/964rz) and longitudinal engagement (https://osf.io/c6azs). *SI Appendix*, Table S2 provides demographic information for the sample of psychologically threatened introductory physics students who enrolled in our RCT.

As preregistered, we examined effects of mindfulness on momentary and longitudinal psychological threat using mixed linear models (MLMs). Together, these preregistered analyses allowed us to understand the duration of training effects on psychological threat. Analyses using momentary threat data examine the immediate impact of the training whereas those using longitudinal threat data investigate its enduring impact. Analyses for both momentary and longitudinal threat were conducted using R 4.4.1 (41) with *lme4* (42) and *r2mlm* (43) packages. When momentary threat was the Level 1 outcome, condition (mindfulness training vs. audiobook control) was the Level 2 fixed effect and the Level 1 intercept was modeled as a random effect, using variance components covariance structure. Time did not moderate the reported effects of the intervention on momentary threat (*P* = 0.65; see *SI Appendix* for details). When longitudinal threat was the Level 1 outcome, assessment (baseline, posttest, 2-wk, and 3-mo follow-up) was a fixed, categorical Level 1 effect. This model also included condition as a fixed Level 2 effect and the Level 1 intercept as a random effect, using variance components. Models were specified using full information maximum likelihood.

Next, we used *car* (44) and *rstatix* (45) packages to fit three separate repeated measures multivariate analysis of covariance (RM-MANCOVA) models testing the effects of mindfulness on the three prespecified clusters of physics engagement outcomes across time (baseline, posttest, 2-wk, and 3-mo follow-up). Significant multivariate condition × assessment interactions were probed using univariate ANCOVAs, with Bonferroni corrections for multiple comparisons (the use of Bonferroni corrections was specified in our preregistration plan and provides the most conservative test of our hypotheses; however, analyses remain significant, in the same direction, and of similar magnitude without correcting for multiple comparisons). As preregistered, we grouped engagement constructs into three clusters based on theory and predicted sensitivity to mindfulness training. Cluster 1 included physics self-efficacy (46), anxiety (47), belonging (48), and performance avoidance goals (49). Cluster 2 included physics identity (48, 50, 51), failure mindset (52), intelligence mindset (24), effort (53), and use of metacognitive study strategies (53, 54). Cluster 3 included physics interest (24, 46, 48, 55), physics value (48, 55), mastery approach goals (49), performance approach goals (49), physics help-seeking behavior (53), and cognitive study strategy use (54). See OSF for full measure items and response scales.

Finally, we investigated whether reduced psychological threat served as an indirect effect variable between mindfulness training and physics engagement using SEM path analyses in *lavaan* (56). Each model used condition as the predictor, a latent psychological threat variable as the mediator, and various engagement constructs measured at 2-wk follow-up assessment as dependent variables (only those engagement constructs that were predicted by mindfulness in univariate ANOVAs described previously were used as outcomes in indirect effect analyses). The latent mediator variable included 7 indicators of threat (6 momentary assessments and posttest assessment survey). Models specified 10,000 bootstrapped samples, full information maximum likelihood, and bias-corrected 95% CI.

As preregistered, our covariates for all reported analyses were self-reported gender (0 = women and nonbinary students, 1 = men), semester week in which the participant began the study, and semester of study enrollment. In indirect effect analyses, we added covariates of baseline threat and the baseline physics engagement construct being analyzed. MANCOVA analyses incorporated baseline engagement into the models directly, rather than as covariates. Analyses remain significant, in the same direction, and of similar magnitude when covariates are not included.

## Results

### Distribution of Psychological Threat in Introductory Physics.

Course-wide, psychological threat was prevalent among introductory physics students who completed the screening survey. Indeed, 50.42% of students reported psychological threat, meaning they perceived the demands of physics to outweigh their coping resources. Moreover, the average threat score (*M* = 0.26, *SD* = 1.55) was above 0, suggesting the sample, on average, felt threatened in physics [*t* (953) = 5.10*, P* < 0.001, 95% CI = [0.16,0.35], *d* = 0.17].

**Fig. 1.**
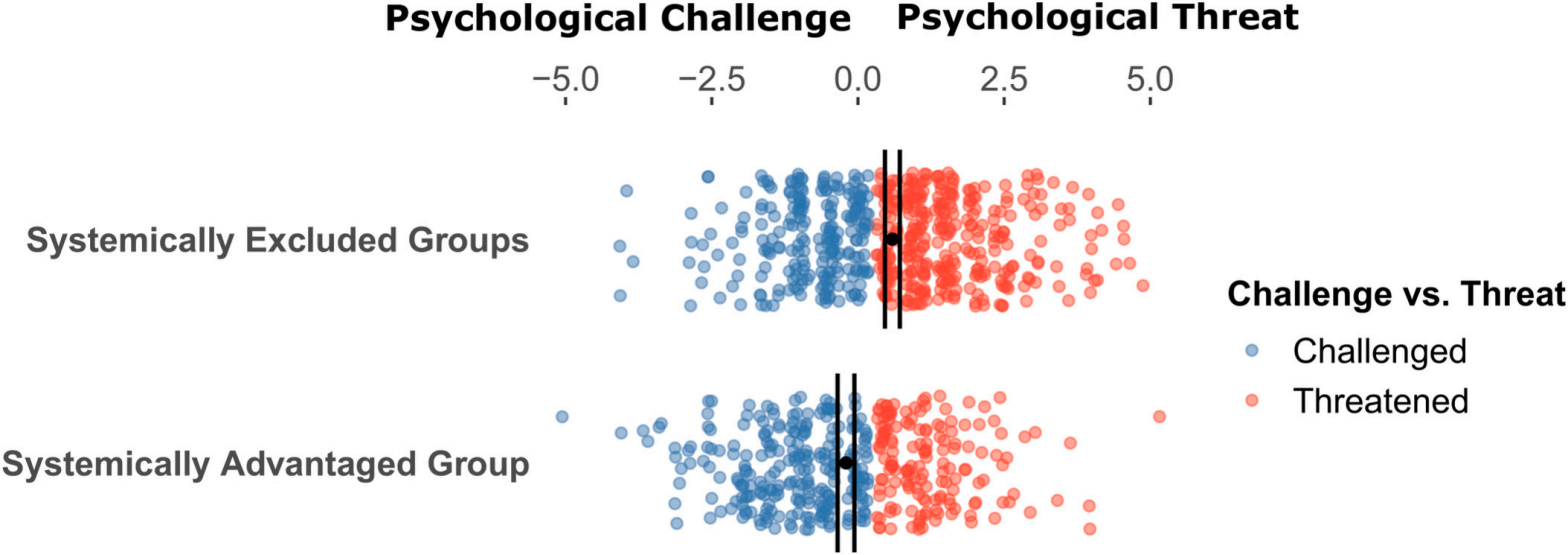
Relationship between identity and psychological threat vs. challenge in physics the systemically excluded group is comprised of students of color across gender identities and white women/nonbinary individuals. The systemically advantaged group includes white men. Error Bars represent 95% CI.

Students’ experiences of threat varied within and across systemically excluded and advantaged groups. As expected, group-level rates of challenge vs. threat experiences differed significantly [*Χ^2^* (1, N = 954) = 37.50, *P* < 0.001, Fig. 1]. Among systemically excluded students, most reported threat (58.95%), though many also reported challenge (41.05%). In contrast, among systemically advantaged students, most reported challenge (61.35%), though a sizable percentage also reported threat (38.65%). As a result, students identified with excluded groups enrolled in our mindfulness experiment at higher rates (72.48%) than those identified with advantaged groups (27.52%; see *SI Appendix*, Tables S8 and S9 for more details). See *SI Appendix* for additional identity-focused distributions of threat and challenge.

### Mindfulness Training Affects Psychological Threat & Engagement.

Mindfulness and audiobook control groups did not differ at baseline on demographics or psychological threat. Full details of baseline equivalence are provided in *SI Appendix*, Table S7. Social identities (i.e., systemically excluded group vs. systemically advantaged group, gender, race/ethnicity) did not moderate any reported effects of the intervention (all *P*s > 0.10; see *SI Appendix* for details of moderation analyses).

### Mindfulness Training Reduces Psychological Threat.

**Fig. 2.**
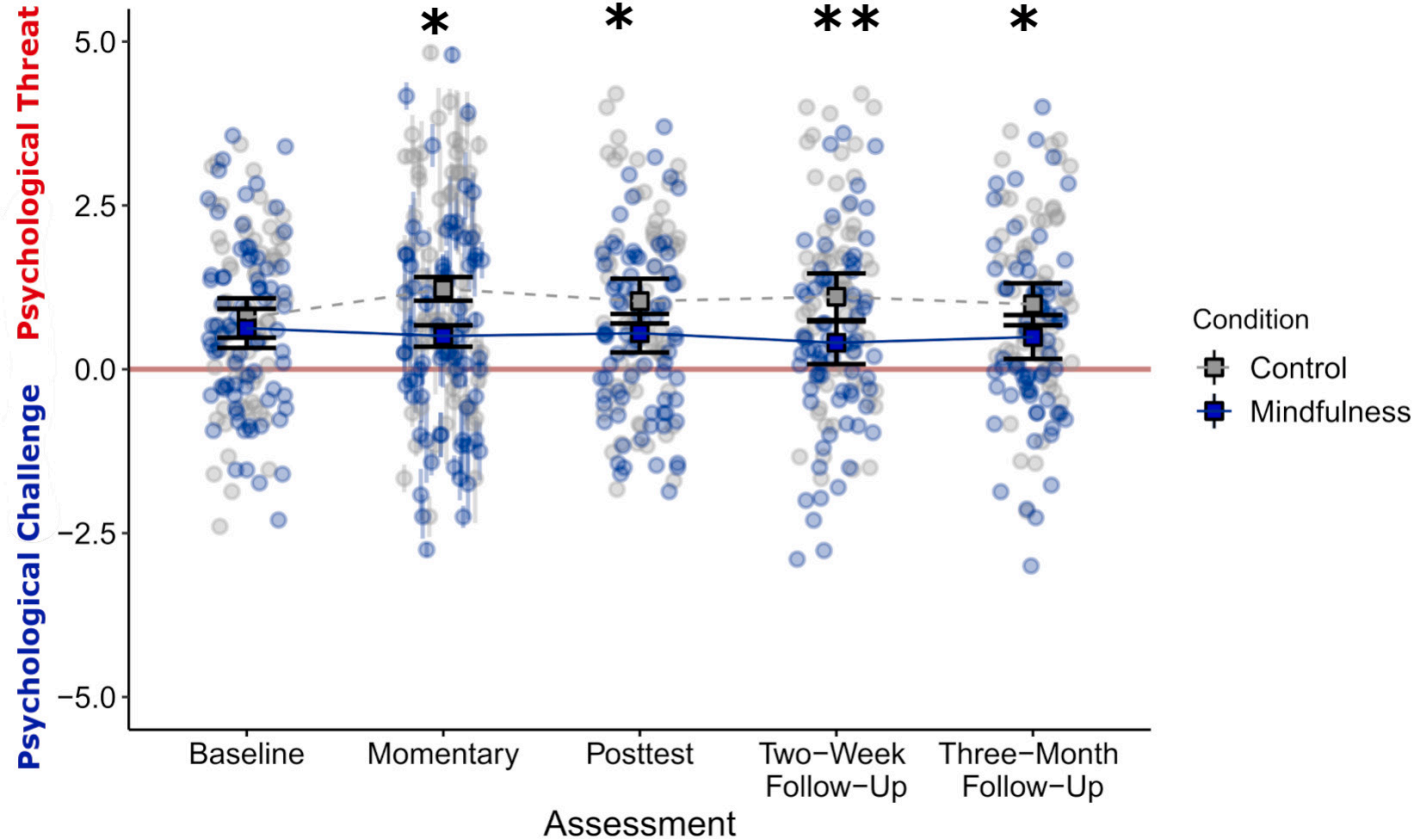
Mindfulness training affects psychological threat across time error bars represent 95% CI. For momentary threat, points and semitransparent lines present means and SEs calculated for each participant (across 6 EMAs), squares with thick black lines show average participant mean by condition and bootstrapped 95% CI. For Baseline, Posttest, and Two-Week and Three-Month follow-ups, points indicate raw participant scores (not model adjusted values) and squares with thick black lines show average by condition and bootstrapped 95% CI. See *SI Appendix*, Table S11 for full pairwise comparison statistics. *P < 0.05, **P < 0.01.

Fig. 2 depicts the effect of mindfulness training on momentary, posttest, and follow-up psychological threat. As predicted, students who received the mindfulness intervention reported lower momentary psychological threat than audiobook control students during training week [*F* (1, 135.07) = 5.66, *P* = 0.019, *pseudo R*^2^ = 0.08, 95% CI = [0.06, 0.23]]. Moreover, while both groups reported similar average threat at baseline, mindfulness participants reported lower psychological threat at posttest, which persisted at 2-wk and 3-mo follow-up [Condition × Assessment Interaction: *F* (3, 417.22) = 4.01, *P* = 0.008, *pseudo R*^2^ = 0.10, 95% CI = [0.08, 0.24]].

To better understand how the intervention shaped participants’ experiences of psychological threat, we examined the effects of mindfulness separately on each of the two components of threat—resources and demands. Mindfulness training students reported greater physics coping resources than audiobook control students during the training week [*F* (1, 135.03) = 8.63, *P* = 0.004, *pseudo R*^2^ = 0.11, 95% CI = [0.08, 0.24]]. These effects endured across time as well: though both groups reported similar average coping resources at baseline, mindfulness participants reported greater resources at posttest, and 2-wk and 3-mo follow-ups [*SI Appendix*, Fig. S9*A*; Condition × Assessment Interaction: *F* (3, 417.64) = 3.18, *P* = 0.024, pseudo *R*^2^ = 0.08, 95% CI = [0.06, 0.22]].

By contrast, mindfulness did not significantly impact perceived physics demands during the training week [*F* (1, 135.17) = 0.92, *P* = 0.340, pseudo *R*^2^ = 0.05, 95% CI = [0.04, 0.17]], or at posttest, 2-wk, and 3-mo follow-up assessments [*SI Appendix*, Fig. S9*B*; Condition × Assessment Interaction: *F* (3, 417.14) = 2.01*, P* = 0.112, pseudo *R*^2^ = 0.12, [0.09, 0.25]]. These results suggest the effect of mindfulness on threat was driven primarily by altering students’ perceptions of their coping resources, not by changing the demands of learning physics.

### Mindfulness Training Promotes Physics Engagement.

We predicted a more robust effect of mindfulness training on Cluster 1 engagement variables compared to Cluster 2 variables. We also anticipated the effect of mindfulness training on Cluster 3 engagement variables, if any, would be small. These predictions were largely supported both in terms of statistical significance and effect size.

Specifically, mindfulness training had a significant and moderate-sized effect on Cluster 1 engagement variables across time [Multivariate Condition × Assessment, Wilks λ = 0.93, *F* (3, 387) = 2.32, *P* = 0.006, η^2^_p_ = 0.02]. The effect of mindfulness on Cluster 2 did not reach our preregistered threshold for significance and had a slightly smaller effect size than that of Cluster 1 [Wilks λ = 0.94, *F* (3, 387) = 1.61, *P* = 0.064, η^2^_p_ = 0.01]. Finally, and as predicted, the training had a nonsignificant and trivial sized effect on Cluster 3 variables [Wilks λ = 0.96, *F* (3, 387) = 0.87, *P* = 0.319, η^2^_p_ < 0.01]. In addition, mindfulness training showed a higher percentage of significant Condition × Assessment interaction effects and a larger average effect size at the univariate level in Cluster 1 variables than in Clusters 2 or 3 variables. *SI Appendix*, Table S12 provides details of all three MANCOVAs and their univariate analyses.

**Fig. 3.**
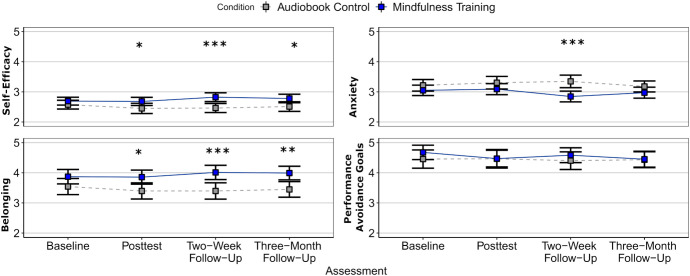
Mindfulness affects Cluster 1 engagement variables across time error bars represent 95% CI. Graph presents raw values averaged within assessment and condition, not model adjusted values. See *SI Appendix*, Table S13 for full pairwise comparison statistics. *P < 0.05, **P < 0.01, ***P < 0.001.

Within Cluster 1, at the univariate level (Fig. 3), mindfulness training (vs. audiobook control) yielded small-to-moderate sized improvements on self-efficacy [Condition × Assessment: *F* (3, 387) = 4.05, *P* = 0.007, η^2^_p_ = 0.03], anxiety [Condition × Assessment: *F* (3, 387) = 4.76, *P* = 0.003, η^2^_p_ = 0.04], and belonging [*F* (3, 387) = 2.49, *P* = 0.060, η^2^_p_ = 0.02]. However, the training did not affect performance avoidance [*F* (3, 387) = 0.98, *P* = 0.402, η^2^_p_ < 0.01].

### Mindfulness-to-Engagement Effects Occur Indirectly Through Psychological Threat.

**Fig. 4.**
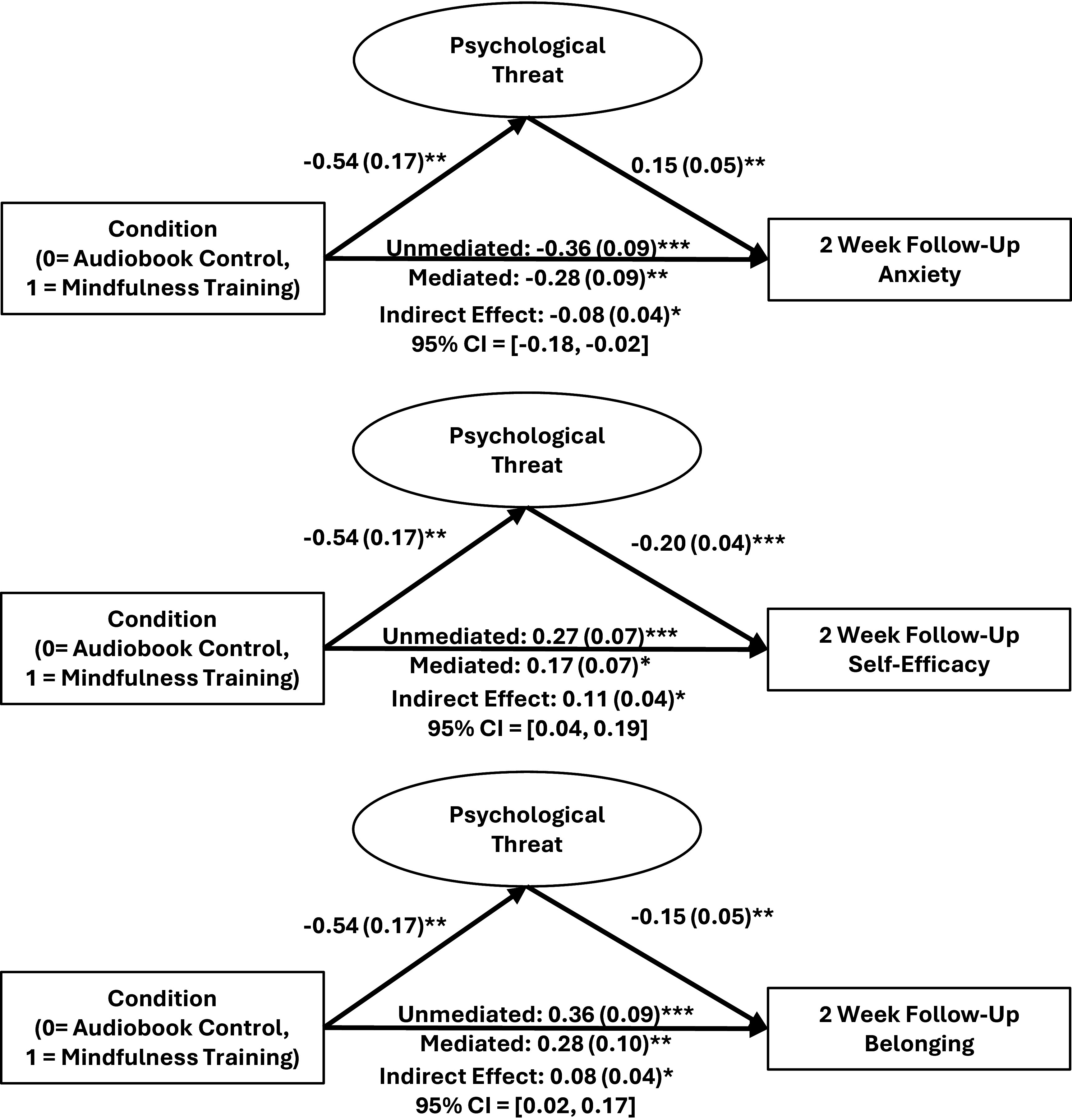
Indirect effects of psychological threat anxiety self-efficacy belonging figures present unstandardized betas with SE in parentheses. Latent psychological threat includes 7 indicators (6 EMA surveys and 1 posttest survey). Covariates include self-reported participant gender, semester week in which participant began the study, study semester enrollment, baseline psychological threat, and baseline anxiety, baseline self-efficacy, or baseline belonging. See *SI Appendix*, Fig. S19 for a model testing the outcome of physics identity. *SI Appendix* also reports models testing engagement outcomes at 3-mo follow-up.

As predicted, mindfulness training was associated with increased physics engagement at the 2-wk follow-up assessment, in part, through a significant indirect effect of psychological threat. Students in the mindfulness training condition (vs. audiobook control) had lower psychological threat during the training week and posttest, which, in turn, was associated with lowered anxiety and greater self-efficacy and belonging 2 wk later (Fig. 4 *A*–*C*).

## Discussion

In this preregistered RCT, brief mindfulness training reduced psychological threat and promoted engagement among undergraduates enrolled in introductory physics. The intervention reduced threat in the moment during the training week and up to 3 mo later. Mindfulness training helped strengthen students’ perceptions of their own coping resources instead of attenuating the demands of learning physics. Mindfulness also fostered greater engagement among constructs expected to most strongly benefit from the training (anxiety, self-efficacy, belonging). As predicted, increases in physics engagement due to mindfulness training were partially explained through reductions in psychological threat.

Beyond testing the effect of mindfulness training, our study also explored patterns of psychological threat in introductory physics classes. Across identity groups, half of students experienced threat (vs. challenge). However, students identified with systemically excluded groups were more likely to experience threat, meaning that our threat-based selection criterion oversampled students from these groups. This recruitment approach might also help explain why social identity did not moderate the impact of mindfulness training: because of group-level differences in the challenge-threat distribution, recruiting only threatened students reduced variation between identity groups for those participating in our study. Nevertheless, the intervention still yielded positive impact overall and conferred these benefits to a greater number of systemically excluded group students who were oversampled as a result of higher group-level threat.

### Implications.

We contribute to clinical science work on mindfulness (5) and the challenge-threat model (9, 10) by applying mindfulness as a tool for helping students optimize their stress. Whereas excessive stress can be harmful in clinical contexts (5), stress in academic settings can confer benefits when viewed as a challenge rather than a threat (10, 26). In our intervention, psychologically threatened students were taught how to use mindfulness to understand, process, and interpret stressful physics experiences in new ways. Specifically, students learned to normalize and deidentify from negative thoughts and emotions, which may have enabled a broader perspective on these difficulties. This broader perspective may have also led students to perceive previously unacknowledged avenues and resources for overcoming their challenges, increasing their overall sense of control over the demands of physics.

Including only psychologically threatened students in our experiment highlights the importance of prioritizing psychological processes over targeting specific identity groups in equity and inclusion-focused STEM interventions. Our approach recognizes that student-level experiences within identity groups vary even as broader contextual factors can exacerbate group-level threat for systemically excluded students. This strategy centers psychological threat without assuming monolithic identity-based experiences or essentializing it within social identities (34). Moreover, it offers potential support for students whose identities are concealable but also systemically excluded (e.g., disability status, sexual orientation).

### Limitations and Future Directions.

Although this confirmatory, preregistered RCT replicates our pilot study results, future replication to explore generalizability to other intervention contexts remains important. The sample from an R1 university in our study limits generalizability to other universities and non-WEIRD populations. Additionally, many physics faculty in the department where we conducted this experiment were open to curriculum innovation and eager for research engagement, perhaps suggesting particularly fertile “soil” for intervention “seeds” (57). Finally, replicating this work in other high-demand STEM courses in the United States can help elucidate the generalizability of these findings to other STEM contexts and departments.

Screening and recruiting students experiencing psychological threat also limits generalizability of these findings to the broader population of introductory physics students. Additionally, our participants were motivated to engage in an intensive research study; which may not be representative of the large population of introductory physics students. Yet, this recruitment strategy has practical advantages rooted in prior work (5): we expected threatened students would be motivated to engage in the sustained effort mindfulness training requires (39). Moreover, psychological threat was a prevalent feature in introductory physics, reported by half of screening survey students. Targeting our intervention toward these students addresses a common part of the physics experience, providing an opportunity to help a substantial portion of students shift how they engage with and interpret their physics coursework. Despite these advantages, the question of whether and how mindfulness training might benefit students reporting psychological challenge cannot be addressed in this work and, thus, exploring whether there are positive, negative, or null effects for these students yields an interesting direction for future work.

Although audiobook controls have been used in prior mindfulness intervention studies (58–61), as a passive control, it cannot directly rule out alternate explanations for these results (e.g., increases in relaxation). However, evidence that reading and auditory experiences (e.g., podcasts) can also promote well-being and reduce stress (62–64) suggests the effects of our mindfulness training are not merely attributable to changes in relaxation. Testing mindfulness training against active control conditions remains an important next step.

Finally, our intervention targeted students’ own cognitive appraisals, but person-level *and* context-level factors produce psychological threat together. Cultivating student resilience may require acknowledging that people both influence and are influenced by their environment (13, 14). An interesting question then is whether mindfulness training for some students can produce emergent effects for their classmates (as in ref. 65). Other interesting opportunities for building on this work include assessing student perceptions of the context and/or integrating mindfulness in classrooms.

## Conclusion

Many college students experience psychological threat and disengagement in introductory physics. Students leveraged mindfulness training to appraise physics as less threatening which, in turn, promoted student engagement. Thus, our intervention empowered students to remember that they are braver than they believe, stronger than they seem, and smarter than they think (1), supporting resilience and engagement in STEM.

## Method

### Transparency and Ethical Research Statement.

University of Pittsburgh IRB approved this research.

We registered this study as a clinical trial (https://clinicaltrials.gov/study/NCT04589377) on 10/08/2020 before data collection. Hypotheses and analyses were preregistered following data collection and prior to viewing data. We preregistered psychological threat (https://osf.io/964rz) on 3/20/2022 and engagement (https://osf.io/c6azs) on 06/17/2022. Measures, primary data, and analysis scripts are publicly available (https://osf.io/wv6xt/?view_only=c1f8794767164388a720cc3316deb9d6) (66). Copyright restrictions prevent publicly sharing mindfulness training, but it can be provided upon request.

These data are part of a larger project testing whether mindfulness training affects physics engagement, learning, and performance outcomes. This manuscript reports only measures relevant to current hypotheses.

### Recruitment.

We assessed psychological threat among undergraduate students enrolled in calculus-based introductory physics courses (11 sections) at a large, public Mid-Atlantic University using a brief online survey. Students who completed the screening survey (N = 954, age: *M* = 18.50 y, *SD* = 1.41) were entered into a raffle to win one of three $25 gift cards, with separate raffles for each course section. *SI Appendix*, Table S1 provides screening survey demographics and *SI Appendix*, Fig. S1 diagrams participant flow from recruitment through study completion.

### Participants.

Students were eligible to participate if they were enrolled in the first course in the two-course introductory calculus-based physics sequence, reported experiencing psychological threat (10), were at least 18 y old, and fluent in English. Participants (N = 149, age: *M* = 18.78, *SD* = 1.80) who met eligibility criteria and enrolled in the experiment received up to $150 for their participation. *SI Appendix*, Table S2 provides participant demographics.

We aimed to detect a conventionally small-to-medium effect size (d = 0.40) in psychological threat, with 80% power (67), providing a target sample size of 150 participants.

### Procedure.

Primary study activities took place Monday (Day 1) through Friday (Day 5). *SI Appendix*, Fig. S2 presents the study timeline. On Monday (Day 1), small participant groups completed an introductory session with an experimenter. Following a detailed explanation of study activities, procedures, timeline, potential risks and benefits, and compensation structure, participants reviewed and provided informed consent using a secure, University-hosted DocuSign online platform. During the self-paced session, participants completed surveys including measures of challenge-threat and engagement in physics, along with other tasks. Next, participants were randomly assigned to either mindfulness training or an audiobook control. Finally, students listened to their first 20-minute mindfulness audio training lesson or an audiobook (to match for time).

On Tuesday through Thursday (Days 2 to 4), all participants completed Ecological Momentary Assessment (EMA) surveys twice a day (afternoon and evening) assessing psychological threat among other constructs. On these days, those in the mindfulness training also listened to their 20-minute audio training in the afternoon session while those in the audiobook control responded only to EMA surveys.

On Friday (Day 5), students listened to their final mindfulness audio or a second audiobook. All participants then completed posttest assessments of challenge-threat and engagement in physics, along with other tasks.

Finally, participants completed surveys assessing challenge-threat and engagement in physics (and other constructs) 2 wk and 3 mo following posttest.

Overall compliance was high across all assessments, with students completing 96.44% of all assessments. *SI Appendix*, Tables S5-S6 provide detailed information regarding compliance rate and number of responses across the study and by condition.

### Experimental conditions.

#### Intervention development.

We created our intervention to target the specific worries and doubts students commonly experience in introductory physics. We conducted 1-hour interviews with introductory physics students to learn more about their specific concerns and interpretations (68). Students described their experiences navigating difficulties during introductory physics. Common physics demands were social comparison, exam worries, and lack of understanding. We used these discussions to design an intervention guiding students through recognizing and accepting their physics-related emotions and thoughts as normal and temporary.

#### Mindfulness training.

Students assigned to the mindfulness intervention received five 20-minute audio lessons, delivered once a day. Lessons were completed via computer on Days 1 and 5, and smartphone on Days 2 to 4. This training taught students about the mindfulness practice “R.A.I.N.,” which stands for Recognize, Accept, Investigate, and Non-Identify (35, 36, 69). We adapted this practice to help students manage common physics demands (e.g., self-doubt, social comparison) identified in interviews. Each lesson invited students to recall a difficult physics experience and process that event using R.A.I.N. The training guided students through observing their experience-related emotions and thoughts as temporary and normal. Attention to the training was assessed following each lesson (86.84 to 97.33% pass rate across lessons, see *SI Appendix* for more details). Mindfulness training participants completed their afternoon EMA surveys immediately following their daily lesson.

#### Audiobook control.

Students assigned to audiobook control listened to two 20-min audio-delivered short stories on Days 1 [“The Stolen Dream”; (70)] and 5 [“The Bet”; (71)] during baseline and posttest assessments. Attention to the audiobook was assessed following each story (90.28 to 98.61% pass rate across stories).

### Psychological threat.

Participants reported their perceptions of physics demands and coping resources using the stress appraisal questionnaire (adapted from ref. 10). Items, scale, and alphas are provided in *SI Appendix*, Table S3. Average perceived coping resources were subtracted from average perceived situational demands to compute psychological threat. Scores at or below zero indicate challenge. Scores above zero indicated threat. Threat was assessed at all timepoints.

#### Engagement.

Physics engagement was assessed through a variety of measures at baseline (Day 1), posttest (Day 5), and 2-wk and 3-mo follow-ups. *SI Appendix*, Table S4 provides construct names, alphas, and number of items for each engagement construct.

## Supplementary Material

Appendix 01 (PDF)

## Data Availability

Anonymized Measures, primary data, and analysis scripts have been deposited in OSF (https://osf.io/vt4jr/) (66). Some study data available (Copyright restrictions prevent publicly sharing mindfulness audio, but it can be provided upon request.).

## References

[1] Pooh’s Grand Adventure: The Search for Christopher Robin (Walt Disney Home Video, 1997).

[2] N. Dasgupta, M. M. Scircle, M. Hunsinger, Female peers in small work groups enhance women’s motivation, verbal participation, and career aspirations in engineering. Proc. Natl. Acad Sci. U.S.A. **112**, 4988–4993 (2015). 25848061 10.1073/pnas.1422822112 PMC4413283

[3] C. M. Steele, A threat in the air. Am. Psychol. **52**, 613–629 (1997). 9174398 10.1037//0003-066x.52.6.613

[4] S. R. Bishop , Mindfulness: A proposed operational definition. Clin. Psychol. Sci. Pract. **11**, 230–241 (2004).

[5] J. D. Creswell, Mindfulness interventions. Annu. Rev. Psychol. **68**, 491–516 (2017). 27687118 10.1146/annurev-psych-042716-051139

[6] T. S. Samuel, J. Warner, “I Can Math!”: Reducing math anxiety and increasing math self-efficacy using a mindfulness and growth mindset-based intervention in first-year students. Community Coll. J. Res. Pract. **45**, 205–222 (2021).

[7] J. B. Chancey, B. C. Heddy, M. Lippmann, E. Abraham, Using an online-based mindfulness intervention to reduce test anxiety in physics students. J. Cogn. Enhanc. **7**, 128–139 (2023). 10.1007/s41465-023-00261-2 PMC10099027 37363055

[8] W. C. Crone , Cultivating well-being in engineering graduate students through mindfulness training. PLoS ONE **18**, e0281994 (2023). 36947553 10.1371/journal.pone.0281994 PMC10032494

[9] J. Blascovich, “Challenge and threat appraisal” in Handbook of Approach and Avoidance Motivation, A. J. Elliot, Ed. (Psychology Press, New York, NY, 2008), pp. 432–444.

[10] J. P. Jamieson, B. J. Peters, E. J. Greenwood, A. J. Altose, Reappraising stress arousal improves performance and reduces evaluation anxiety in classroom exam situations. Soc. Psychol. Personal. Sci. **7**, 579–587 (2016).

[11] J. P. Jamieson, A. J. Crum, J. P. Goyer, M. E. Marotta, M. Akinola, Optimizing stress responses with reappraisal and mindset interventions: An integrated model. Anxiety, Stress, Coping **31**, 245–261 (2018). 29471669 10.1080/10615806.2018.1442615

[12] J. P. Jamieson, “Challenge and threat appraisals” in Handbook of competence and motivation: Theory and application, A. J. Elliot, C. S. Dweck, D. S. Yeager, Eds. (Guilford Press, New York, NY, 2018), pp. 175–191.

[13] K. M. Kroeper , The recursive cycle of perceived mindset and psychological distress in college. Soc. Psychol. Pers. Sci. **29**, 19485506241247384 (2024).

[14] H. R. Markus, S. Kitayama, Cultures and selves: A cycle of mutual constitution. Perspect. Psychol. Sci. **5**, 420–430 (2010). 26162188 10.1177/1745691610375557

[15] M. C. Murphy, C. M. Steele, J. J. Gross, Signaling threat. Psychol. Sci. **18**, 879–885 (2007). 17894605 10.1111/j.1467-9280.2007.01995.x

[16] E. A. Canning, J. LaCosse, K. M. Kroeper, M. C. Murphy, Feeling like an imposter: The effect of perceived classroom competition on the daily psychological experiences of first-generation college students. Soc. Psychol. Pers. Sci. **11**, 647–657 (2020).

[17] E. A. Canning, K. Muenks, D. J. Green, M. C. Murphy, STEM faculty who believe ability is fixed have larger racial achievement gaps and inspire less student motivation in their classes. Sci. Adv. **5**, eaau4734 (2019). 30793027 10.1126/sciadv.aau4734 PMC6377274

[18] K. Muenks , Does my professor think my ability can change? Students’ perceptions of their STEM professors’ mindset beliefs predict their psychological vulnerability, engagement, and performance in class. J. Exp. Psychol. Gen. **149**, 2119–2144 (2020). 32378957 10.1037/xge0000763

[19] L. Bian, S. J. Leslie, A. Cimpian, Gender stereotypes about intellectual ability emerge early and influence children’s interests. Science. **355**, 389–391 (2017). 28126816 10.1126/science.aah6524

[20] M. Meyer, A. Cimpian, S. J. Leslie, Women are underrepresented in fields where success is believed to require brilliance. Front. Psychol. **6**, 1–12 (2015). 25814964 10.3389/fpsyg.2015.00235 PMC4356003

[21] D. Storage, Z. Horne, A. Cimpian, S. J. Leslie, The frequency of “brilliant” and “genius” in teaching evaluations predicts the representation of women and African Americans across fields. PLoS ONE **11**, 1–18 (2016). 10.1371/journal.pone.0150194 PMC4777431 26938242

[22] L. Bian, S. J. Leslie, M. C. Murphy, A. Cimpian, Messages about brilliance undermine women’s interest in educational and professional opportunities. J. Exp. Soc. Psychol. **76**, 404–420 (2018).

[23] Z. Y. Kalender, E. Marshman, C. D. Schunn, T. J. Nokes-Malach, C. Singh, Framework for unpacking students’ mindsets in physics by gender. Phys. Rev. Phys. Educ. Res. **18**, 010116 (2022).

[24] E. Marshman, Z. Y. Kalender, C. Schunn, T. Nokes-Malach, C. Singh, A longitudinal analysis of students’ motivational characteristics in introductory physics courses: Gender differences. Can. J. Phys. **96**, 391–405 (2018).

[25] S. J. Spencer, C. M. Steele, D. M. Quinn, Stereotype threat and women’s math performance. J. Exp. Soc. Psychol. **35**, 4–28 (1999).

[26] J. P. Jamieson , Reappraising stress arousal improves affective, neuroendocrine, and academic performance outcomes in community college classrooms. J. Exp. Psychol. Gen. **151**, 197–212 (2022). 34292050 10.1037/xge0000893

[27] K. R. Binning , Securing self-integrity over time: Self-affirmation disrupts a negative cycle between psychological threat and academic performance. J. Soc. Issues **77**, 801–823 (2021).

[28] D. K. Sherman , Deflecting the trajectory and changing the narrative: How self-affirmation affects academic performance and motivation under identity threat. J. Pers. Soc. Psychol. **104**, 591–618 (2013). 23397969 10.1037/a0031495

[29] J. A. Fredricks, P. C. Blumenfeld, A. H. Paris, School engagement: Potential of the concept, state of the evidence. Rev. Educ. Res. **74**, 59–109 (2004).

[30] M. T. Wang, J. A. Fredricks, F. Ye, T. L. Hofkens, J. S. Linn, The math and science engagement scales: Scale development, validation, and psychometric properties. Learn. Instr. **43**, 16–26 (2016).

[31] J. A. Gasiewski, M. K. Eagan, G. A. Garcia, S. Hurtado, M. J. Chang, From gatekeeping to engagement: A multicontextual, mixed method study of student academic engagement in introductory STEM courses. Res. High. Educ. **53**, 229–261 (2012). 23503751 10.1007/s11162-011-9247-y PMC3596160

[32] M. L. Bernacki, T. J. Nokes-Malach, V. Aleven, Examining self-efficacy during learning: Variability and relations to behavior, performance, and learning. Metacogn. Learn. **10**, 99–117 (2015).

[33] G. M. Walton, G. L. Cohen, A brief social-belonging intervention improves academic and health outcomes of minority students. Science. **331**, 1447–1451 (2011). 21415354 10.1126/science.1198364

[34] A. B. Diekman, E. K. Clark, A. L. Belanger, Finding common ground: Synthesizing divergent theoretical views to promote women’s STEM pursuits. Soc. Issues Policy Rev. **13**, 182–210 (2019).

[35] T. Brach, True Refuge: Finding Peace and Freedom in Your Own Awakened Heart (Bantam Books, New York, NY, 2013).

[36] J. A. Brewer, The Craving Mind: From Cigarettes to Smartphones to Love? Why We Get Hooked and How We Can Break Bad Habits (Yale University Press, New Haven, CT, 2017).

[37] J. D. Creswell, L. E. Pacilio, E. K. Lindsay, K. W. Brown, Brief mindfulness meditation training alters psychological and neuroendocrine responses to social evaluative stress. Psychoneuroendocrinology **44**, 1–12 (2014). 24767614 10.1016/j.psyneuen.2014.02.007

[38] F. C. M. Geisler, M. N. Bechtoldt, N. Oberländer, M. Schacht-Jablonowsky, The benefits of a mindfulness exercise in a performance situation. Psychol. Rep. **121**, 853–876 (2018). 29298588 10.1177/0033294117740135

[39] J. Kabat-Zinn, Full Catrosphe Living: Using The Wisdom of Your Body and Mind to Face Stress, Pain, and Illness (Bantam Books, 2013).

[40] M. Cikara, J. E. Martinez, N. A. Lewis, Moving beyond social categories by incorporating context in social psychological theory. Nat Rev Psychol. **1**, 537–549 (2022).

[41] R Core Team, R: A Language and Environment for Statistical Computing [Internet]. (R Foundation for Statistical Computing, 2024), https://www.R-project.org/. Accessed 19 December 2025.

[42] D. Bates, M. Maeschler, B. Bolker, S. Walker, Fitting linear mixed-effects models using {lme4}. J. Stat. Softw. **67**, 1–48 (2015).

[43] M. Shaw, J. D. Rights, S. K. Sterba, J. K. Flake, r2mlm: An R package calculating R-squared measures for multilevel models. Behav. Res. Methods **55**, 1942–1964 (2023). 35798918 10.3758/s13428-022-01841-4

[44] J. Fox, S. Weisberg, An R Companion to Applied Regression. Third Edition (Sage, Thousand Oaks, CA, 2019).

[45] A. Kassambara, rstatix: Pipe-friendly framework for basic statistical tests [Internet] (2019), p. 0.7.2. https://CRAN.R-project.org/package=rstatix. Cited 14 April 2025.

[46] S. M. Glynn, P. Brickman, N. Armstrong, G. Taasoobshirazi, Science motivation questionnaire. II: Validation with science majors and nonscience majors. J. Res. Sci. Teach. **48**, 1159–1176 (2011).

[47] D. R. Hopko, R. Mahadevan, R. L. Bare, M. K. Hunt, The abbreviated math anxiety scale (AMAS): Construction, validity, and reliability. Assessment **10**, 178–182 (2003). 12801189 10.1177/1073191103010002008

[48] Z. Y. Kalender, E. Marshman, C. D. Schunn, T. J. Nokes-Malach, C. Singh, Gendered patterns in the construction of physics identity from motivational factors. Phys. Rev. Phys. Educ. Res. **15**, 020119 (2019).

[49] A. J. Elliot, K. Murayama, On the measurement of achievement goals: Critique, illustration, and application. J. Educ. Psychol. **100**, 613–628 (2008).

[50] Z. Hazari, R. H. Tai, P. M. Sadler, Gender differences in introductory university physics performance: The influence of high school physics preparation and affective factors. Sci. Educ. **91**, 847–876 (2007).

[51] Z. Hazari, G. Sonnert, P. M. Sadler, M. C. Shanahan, Connecting high school physics experiences, outcome expectations, physics identity, and physics career choice: A gender study. J. Res. Sci. Teach. **47**, 978–1003 (2010).

[52] K. Haimovitz, C. S. Dweck, Parents’ views of failure predict children’s fixed and growth intelligence mind-sets. Psychol. Sci. **27**, 859–869 (2016). 27113733 10.1177/0956797616639727

[53] P. R. Pintrich, D. A. F. Smith, T. Garcia, W. J. McKeachie, A Manual for the Use of the Motivated Strategies for Learning Questionnaire (MSLQ) (University of Michigan, 1991).

[54] C. D. Zepeda, T. J. Nokes-Malach, Metacognitive study strategies in a college course and their relation to exam performance. Mem. Cogn. **49**, 480–497 (2021). 10.3758/s13421-020-01106-5 33150557

[55] J. Chung, M. A. Cannady, C. Schunn, R. Dorph, M. Bathgate, “Measures technical brief: Fascination in Science” (Report No. 3.2-20160331, Activation Lab, 2016).

[56] Y. Rosseel, Lavaan: An R package for structural equation modeling. J. Stat. Softw. **48**, 1–36 (2012).

[57] G. M. Walton, D. S. Yeager, Seed and soil: Psychological affordances in contexts help to explain where wise interventions succeed or fail. Curr. Dir. Psychol. Sci. **29**, 219–226 (2020). 32719574 10.1177/0963721420904453 PMC7384695

[58] M. Economides, J. Martman, M. J. Bell, B. Sanderson, Improvements in stress, affect, and irritability following brief use of a mindfulness-based smartphone app: A randomized controlled trial. Mindfulness **9**, 1584–1593 (2018). 30294390 10.1007/s12671-018-0905-4 PMC6153897

[59] N. Eisenbeck, C. Luciano, S. Valdivia-Salas, Effects of a focused breathing mindfulness exercise on attention, memory, and mood: The importance of task characteristics. Behav. Change **35**, 54–70 (2018).

[60] F. Zeidan, S. K. Johnson, B. J. Diamond, Z. David, P. Goolkasian, Mindfulness meditation improves cognition: Evidence of brief mental training. Conscious. Cogn. **19**, 597–605 (2010). 20363650 10.1016/j.concog.2010.03.014

[61] A. Sparacio , Self-administered mindfulness interventions reduce stress in a large, randomized controlled multi-site study. Nat. Hum. Behav. **8**, 1716–1725 (2024). 38862815 10.1038/s41562-024-01907-7 PMC11420060

[62] B. Dreer, Fostering well-being over the radio? An empirical study investigating the effects of an audio podcast-based intervention program on student teachers’ well-being. Int. J. Community Well-Being **4**, 603–623 (2021).

[63] K. Hodgson, R. Thomson, What do medical students read and why? A survey of medical students in Newcastle-upon-Tyne England. Med. Educ. **34**, 622–629 (2000). 10964209 10.1046/j.1365-2923.2000.00542.x

[64] M. V. R. Kustritz, Yoga and leisure reading for stress management and wellness at a veterinary medical college. J. Am. Vet. Med. Assoc. **258**, 948–951 (2021). 33856873 10.2460/javma.258.9.948

[65] J. T. Powers , Changing environments by changing individuals: The emergent effects of psychological intervention. Psychol. Sci. **27**, 150–160 (2016). 26671909 10.1177/0956797615614591

[66] T. M. Benson-Greenwald , Data & scripts for main text/supplemental analyses: Mindfulness reduces psychological threat and increases engagement in college physics. OSF. http://osf.io/vt4jr/. Deposited 8 September 2025.

[67] F. Faul, E. Erdfelder, A. Buchner, A. G. Lang, Statistical power analyses using G*Power 3.1: Tests for correlation and regression analyses. Behav. Res. Methods **41**, 1149–1160 (2009). 19897823 10.3758/BRM.41.4.1149

[68] A. Pelakh , Thematic analysis of student perceptions of resources and demands experienced in introductory physics. arXiv [Preprint] (2025). https://arxiv.org/abs/2502.14692 (Accessed 3 March 2025).

[69] The RAINDROP Acronym [Internet] (RAIN: The Nourishing Art of Mindfulness Inquiry). https://learn.tricycle.org/courses/rain/lectures/22518332. Accessed 4 September 2025.

[70] R. Le Gallienne, The Stolen Dream (Redwood Audiobooks, Mendocino, CA, 1912).

[71] A. Chekhov, The Bet (Redwood Audiobooks, Mendocino, CA, 1889).

