# Supplementary material for: Building courage, strength, and knowledge: Mindfulness training reduces psychological threat and increases engagement in college physics
Source: Proc Natl Acad Sci U S A. 2026 Apr 6;123(15):e2521857123. doi: 10.1073/pnas.2521857123 (PMC13079943; doi:10.1073/pnas.2521857123)
Supplement: Supplementary file 1 — Appendix 01 (PDF) [file pnas.2521857123.sapp.pdf]

Building Courage, Strength, Knowledge: Mindfulness Training Reduces Psychological Threat in College Physics

Supplemental Material

Supplement Table of Contents

Full Acknowledgements 5
Explanation of Systemically Excluded Groups vs. Systemically Advantaged Group 6
Additional Methodological Details: Screening Survey & Pre-Registered RCT 7
Table S1. Screening Survey Demographics (N = 954) 7
Table S2. RCT (N = 149) Demographic Information 8
Figure S1. Participant Flow (CONSORT) Diagram 9
Figure S2. Timeline of primary study activities. 10
Table S3. Psychological Threat vs. Challenge Items & Alphas 11
Table S4. Engagement Measurement Alphas 12
Table S5. Compliance Rates 13
Table S6. Number of Responses at Each Assessment 14
Baseline Equivalence 15
Table S7. RCT Baseline Equivalence 15
Screening Survey Results 16
Full Screening Survey Sample 16
Figure S3. Introductory Physics Challenge-Threat Distribution 16
Threat Distribution Across Identity Groups 16
Figure S4. Screening Survey Threat by Identification 17
Table S8. Screening vs. Enrolled Participant Proportion by Demographic Category 18
Table S9. Descriptive Statistics for Psychological Threat across Demographic Category 19
Figure S5. Threat Distribution by Racial & Gender Identification 20
Enrolled Participants at Screening 21
Figure S6. Threat Distribution Across Identity Groups Among Enrolled Participants (N = 149) 21
Not Enrolled and Ineligible Students 22
Figure S7. Threat Distribution Across Identity Groups Among Not Enrolled Students (N = 279) 22
Figure S8. Threat Distribution Across Identity Groups Among Ineligible Students (N = 526) 24
Table S10. Screening & Not Enrolled, Ineligible by Demographic Category 25
Additional EMA Analyses 26
EMA Robustness Analyses – Without Covariates 26
EMA Moderation Analyses 26
Additional Longitudinal Threat Analyses 29

Figure S9. Long Term Effects of Mindfulness Training on Resource & Demand Appraisals 30
Table S11. Pairwise Contrasts for Longitudinal Threat & Resources 31
Longitudinal Robustness Analyses – without covariates 31
Longitudinal Moderation Analyses 32
Condition × Time × Identity Classification 32
Condition × Time × Racial Identification 32
Condition × Time × Gender Identification 33
Additional Longitudinal Engagement Analyses 33
Table S12. Condition × Assessment Effects on Engagement: Multivariate & Univariate 34
Table S13. Cluster 1 Engagement Pairwise Comparisons 35
Figure S10. Effect of Condition on Physics Identity Across Time 36
Table S14. Pairwise Comparisons for Condition × Assessment Effect on Physics Identity 37
Latent Threat Measurement Model Details 38
Additional Indirect-Effects Analyses 38
Figure S11. Indirect Effects of Psychological Threat 39
Figure S12. 3-Month Follow-Up Self-Efficacy and Physics Identity Indirect Effect Models 39
Figure S13. 3-Month Follow-Up Anxiety and Belonging Indirect Effect Models 40
Pilot Study 41
Screening Survey & Experiment Sample Demographic Information 41
Table S15. Pilot Sample Demographic Information (N = 27) 41
Table S16. Pilot Screening Survey Demographic Information (N = 127) 42
Table S17. Pilot Study Baseline Equivalence 43
Pilot: Full Screening Survey Sample 44
Figure S14. Introductory Physics Challenge-Threat Distribution 44
Pilot Study: Screening Survey Threat by Racial & Gender Identification 44
Figure S15. Screening Survey Threat by Identification (N = 127) 45
Table S18. Screening vs. Enrolled Participant Proportion by Demographic Category 45
Table S19. Descriptive Statistics for Psychological Threat across Demographic Category 46
Figure S16. Threat Distribution by Racial and Gender Identification 47
Enrolled Participants at Screening 48
Figure S17. Pilot Study Enrolled Participants (N = 27) 48
Not Enrolled and Ineligible Students 49
Figure S18. Distribution of Threat Among Not Enrolled Students (N = 56) 49

Figure S19. Threat Distribution Across Identity Groups Among Ineligible Students (N = 44) 50
Table S20. Screening & Not Enrolled, Ineligible by Demographic Category 51
Pilot EMA Analyses 52
Momentary Threat, Resources, & Demands 52
EMA Robustness Analyses – Without Covariates 52
EMA Moderation Analyses 53
Condition × Time 53
Condition × Analysis Classification 53
Condition × Racial Identification 54
Condition × Gender Identification 54
Pilot Longitudinal Analyses 55
Main Analyses 55
Figure S20. Long Term Effects of Mindfulness Training on Psychological Threat 55
Figure S21. Long Term Effects of Mindfulness Training on Resource & Demand Appraisals 56
Table S21. Pairwise Contrasts for Longitudinal Resources 57
Longitudinal Robustness Analyses – without covariates 57
Longitudinal Moderation Analyses 58
Condition × Time × Analysis Classification 58
Condition × Time × Racial Identification 58
Condition × Time × Gender Identification 58

Full Acknowledgements

We thank Jessica Morey for co-designing and recording the mindfulness lessons. We are also grateful to the many introductory physics instructors who supported recruitment efforts across multiple semesters. We thank Scott Fraundorf for statistical consulting and Chandralekha Singh for her support of the project. We further appreciate the feedback and guidance provided by members of the National Science Foundation advisory board, David Creswell, Zahra Hazari, Jeremy Jamieson. We thank the many other students and staff who contributed to the broader project, including PhD students Shana DeVlieger, Angela Griffo, Nabila Jamal-Orozco, and Therese Schnettler; master's students Kristin Hinshaw, Sai Aishwarya Karanam; project coordinators Tyree Langley, Dalia Maeroff, and McNoah Vinoya; and undergraduate research assistants Amy Adelman, Jordann Antoan, Alyssa Chomitzky, Jovan Corrales, Brandon Baun, Nicole El Hayek, Jessica Giuffrida, Irvin Li, Keely Lombardi, Gillian Murphy, Connor McGrath, Brennon Paik, Austin Rosenkranz, Kayla Tupper, Zihui Zhang, and Alexi Zukas.

Explanation of Systemically Excluded Groups vs. Systemically Advantaged Group

Systemically excluded groups include any social identity/demographic group that has been systemically denied access to power and structures (e.g., higher education; 1). This structural denial of access includes systematic prejudice and discrimination due to race, ethnicity, gender, or other social identities. This term stands in contrast to terms such as underrepresented groups/underrepresented minorities which fail to recognize the structural barriers to access that contribute to numerical representation. Moreover, it allows for the consideration of systemic inequities even in contexts/cases where groups are numerically overrepresented but still face prejudice and discrimination (e.g., Asian/Asian American students in STEM contexts in the U.S.). Notably, this term implies only the commonality of systemic exclusion and does not make claims that such denials of power/access have been enacted or experienced similarly across particular identities. The systemically advantaged group is the cluster of social identities that have held power in and had consistent access to these same structures.

In the current research, students of color and white women/non-binary individuals are categorized as systemically excluded groups. Students who identified as white men are categorized as the systemically advantaged group.

Additional Methodological Details: Screening Survey & Pre-Registered RCT

Table S1. Screening Survey Demographics (N = 954)

	N	% of sample
**Racial and Ethnic Identification** *(Participants could select more than one):*[a]		
American Indian/Native American/Alaskan Native	4	0.42%
Asian/Indian/Pacific Islander	205	21.49%
Black/African American	45	4.72%
Hispanic/Latine	49	5.14%
Middle Eastern/North African	13	1.36%
White	702	73.58%
Students Who Selected Multiple Identities	69	7.23%
Asian/Indian/Pacific Islander, White		
Asian/Indian/Pacific Islander, Black/African American		
Asian/Indian/Pacific Islander, Hispanic/Latine		
American Indian/Native American/Alaskan Native, Black/African American, Black/African American, Hispanic/Latine		
American Indian/Native American/Alaskan Native, Black/African American, Hispanic/Latine, White		
Black/African American, White		
**Racial and Ethnic Classification**[b]		
Students of Color	311	32.60%
White Students	643	67.40%
**Gender Identification**		
Women	371	38.90%
Non-binary	6	0.73%
Questioning	1	0.10%
Trans man	1	0.10%
Men	572	59.96%
Not provided	3	0.31%
**Analysis Classification**		
Systemically Excluded Groups[c]	553	57.97%
Systemically Advantaged Group[d]	401	42.03%

Note. [a]*Frequencies for racial and ethnic identification sum to greater than 954 (100%) of the sample because participants were able to select multiple race and ethnicity categories. Some participants chose to self-describe.*
[b]*Students of Color includes students who select multiple identities, one of which is not white. For this study, Middle Eastern/North African students are categorized as Students of Color and not White Students. Although this categorization is not universally agreed upon, it reflects how Middle Eastern/North African people are identified as not White, both by Middle Eastern/North African people and White people in the U.S.* (2)
[c]*Systemically excluded groups includes all students of color (any gender), white women, and white non-binary people.*
[d]*Systemically advantaged group includes white men.*

Table S2. RCT (N = 149) Demographic Information

	N	*% of sample*
**Racial and Ethnic Identification** (*Participants could select more than one*)[a]		
American Indian/Native American/Alaskan Native	2	1.34%
Asian/Indian/Pacific Islander	36	24.16%
Black/African American	11	7.38%
Hispanic/Latine	7	4.70%
Middle Eastern/North African	5	3.36%
White	114	76.51%
Students Who Selected Multiple Identities	24	16.11%
American Indian/Native American/Alaskan Native, Black/African American, Hispanic/Latine, White		
American Indian/Native American/Alaskan Native, White		
Asian/Indian/Pacific Islander, Black/African American		
Asian/Indian/Pacific Islander, White		
Black/African American, Hispanic/Latine		
Black/African American, White		
Hispanic/Latine, White		
Middle Eastern/North African, White		
**Racial and Ethnic Classification**		
Students of Color[b]	56	37.58%
White Students	93	62.42%
**Gender Identification**		
Women	81	54.36%
Non-Binary	2	1.34%
Men	66	44.30%
**Analysis Classification**		
Systemically Excluded Groups[c]	108	72.48%
Systemically Advantaged Group[d]	41	27.52%

Note. [a]*Frequencies for racial and ethnic identification sum to greater than 149 (100% of the sample) because participants were able to select multiple race and ethnic categories. Some participants chose to self-describe.*
[b]*Students of Color includes students who select multiple identities, one of which is not white. For this study, Middle Eastern/North African students are categorized as Students of Color and not White Students. Although this categorization is not universally agreed upon, it reflects how Middle Eastern/North African people are identified as not White, both by Middle Eastern/North African people and White people in the U.S.* (2).
[c]*Systemically excluded group classification includes all students of color (any gender), white women, and white non-binary people.*
[d]*Systemrically advantaged group classification includes white men.*

Figure S1. Participant Flow (CONSORT) Diagram

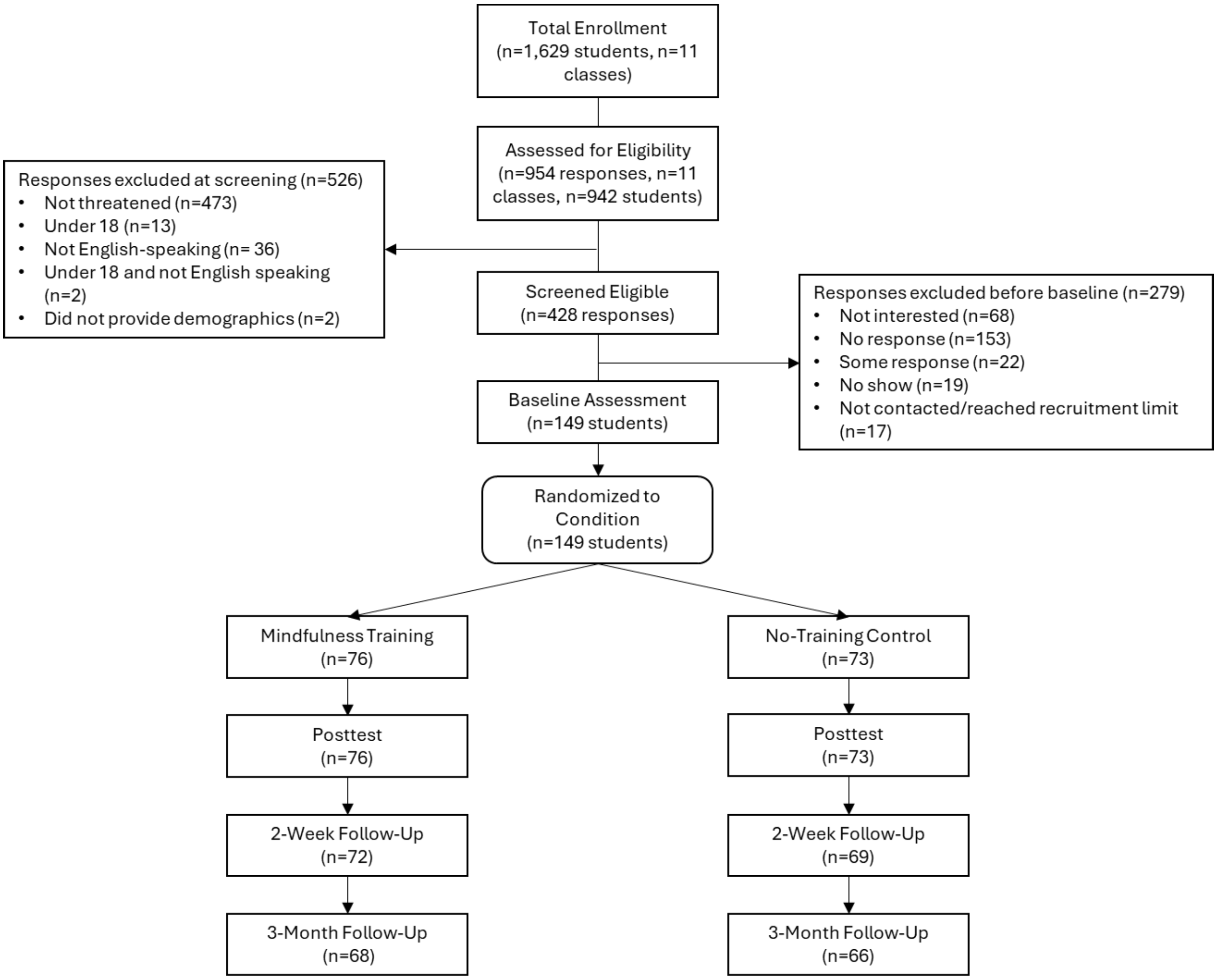

Note. Some students provided multiple responses to the screening survey because they enrolled in Physics 1 in more than one semester. We provide the total number of responses through the screening process and then refer to individual students starting with the baseline assessment.

Figure S2. Timeline of primary study activities.

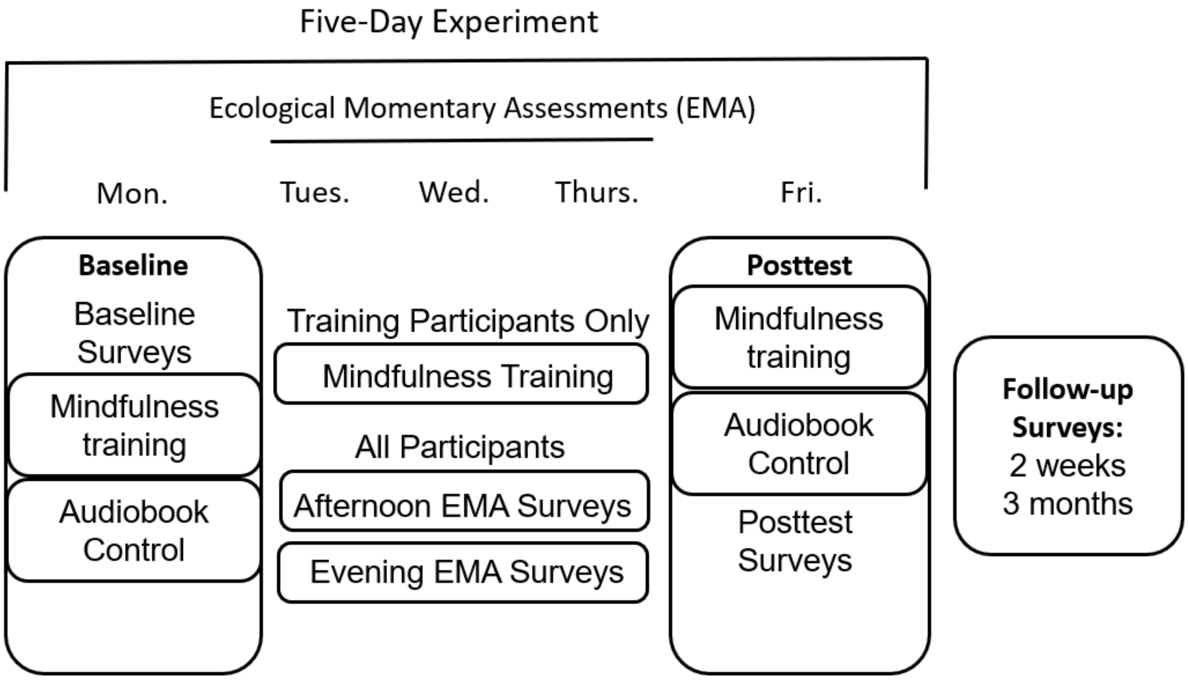

Note. See OSF page for a comprehensive description of the entire project (https://osf.io/2jfvn)

Table S3. Psychological Threat vs. Challenge Items & Alphas

		Demands	Resources
		Working on physics is very demanding.*	I feel that I have the abilities to succeed on my physics work.*
		My physics work is very stressful.*	I'm the kind of person that does well on physics work.*
		Poor performance on physics work would be very distressing for me.	It is very important to me that I perform well on physics work.
		I think physics work represents a threat to me.	I expect to perform well on physics work
		I am uncertain about how I will perform in physics.	I view physics work as a positive challenge.
		My physics work will take a lot of effort to complete.	–
**α**	***4-items***	0.70-0.90	0.74-0.93
	11-items	0.74-0.75	0.78-0.86

Note. Items marked with an asterisk were used in the 4-item version of scale during the Screening Survey and EMA surveys. EMA version of items added the words "Right now," before the statement.

Table S4. Engagement Measurement Alphas

	α	# of Items
Cluster 1		
Self-Efficacy	.75 - .86	5
Anxiety	.84 - .89	8
Belonging	.87 - .90	5
Performance Avoidance Goals	.90 -.95	3
Cluster 2		
Physics Identity	.88 - .92	4
Failure Mindset	.88 - .94	3
Intelligence Mindset	.81 - .86	4
Effort	.74 - .84	4
Metacognitive Study Strategy Use	.69 - .78	7
Cluster 3		
Physics Interest	.86 - .91	5
Physics Value	.90 - .92	4
Mastery Approach Goals	.86 - .95	3
Performance Approach Goals	.82 - .94	3
Physics Help-Seeking Behavior	.65 - .68	4
Cognitive Study Strategy Use	.71 - .86	6

Note. All engagement items and response scales are provided on our OSF (see main text for link).

Table S5. Compliance Rates

		Condition		Test for Compliance Differences			
	Average	***Mindfulness***	***Control***	***t***	***df***	***p-value***	***95% CI***
**All Assessments**	96.44%	96.30%	96.60%	0.19	147	.847	[-.02, .03]
Long-Term	96.10%	96.10%	96.20%	0.10	147	.924	[-.04, .04]
Momentary	96.60%	96.50%	96.80%	0.18	147	.858	[-.03, .04]

Note. Long-term refers to baseline, post-test, 2-Week Follow-Up, and 3-Month Follow-Up surveys. Momentary refers to the 6 EMA surveys participants completed in the afternoon and evening on days 2 (Tuesday) through 4 (Thursday) of the experiment. One participant did not complete any of the momentary assessments but did complete all long-term surveys.

Table S6. Number of Responses at Each Assessment

		Responses by Condition	
Long-Term Assessments	**Total Responses**	***Mindfulness***	***Control***
Baseline	149	76	73
Post-test	148	76	72
2-Week Follow-Up	140	72	68
3-Month Follow-Up	134	68	66
Momentary Assessments			
EMA 1	145	75	70
EMA 2	147	75	72
EMA 3	146	75	71
EMA 4	142	70	72
EMA 5	143	73	70
EMA 6	140	72	68

Note. For each assessment, the table provides the total number of responses and then the number broken out by condition. The relatively consistent sample size across assessments highlights the high compliance provided in Table S5.

Baseline Equivalence

Table S7 demonstrates successful random assignment to condition. Participants in mindfulness training and audiobook control conditions did not differ significantly at baseline assessment.

Table S7. RCT Baseline Equivalence

	Mindfulness	Control	Test	*df*	*p*	*95% CI*
**Group Size**	76	73				
**Demographics**						
Age	18.76 (2.02)	18.78 (1.56)	T	147	.851	[-0.63, 0.52]
Year in School	1	1	X^2	4	.430	N/A
Study Semester Enrollment	Spring 21	Spring 21	X^2	2	.994	N/A
Semester Week of Enrollment	9.13 (2.82)	9.04 (2.82)	T	147	.844	[-1.00, 0.82]
Participant Gender			X^2	1	.912	N/A
Women & Non-Binary	42	41				
Men	34	32				
Racial/Ethnic Identification			X^2	1	.245	N/A
Students of Color	32	24				
White Students	44	49				
Analysis Classification			X^2	1	.738	N/A
Systemically Excluded Groups	56	52				
Systemically Advantaged Group	20	21				
**Psychological Threat**	0.63 (0.15)	0.78 (0.15)	T	147	.459	[-0.26, 0.58]
Demands	4.75 (0.69)	4.81 (0.66)	T	147	.544	[-0.15, 0.29]
Resources	4.12 (0.77)	4.03 (0.80)	T	147	.480	[-0.34, 0.16]

Note: For Age, Semester Week of Enrollment, Threat, Demands, and Resources, we provide means with standard deviations in parentheses. For Year in School and Study Semester Enrollment, we provide the modal response (e.g., 1st year students). All other variables provide frequencies.

Screening Survey Results

Full Screening Survey Sample

Figure S3. Introductory Physics Challenge-Threat Distribution

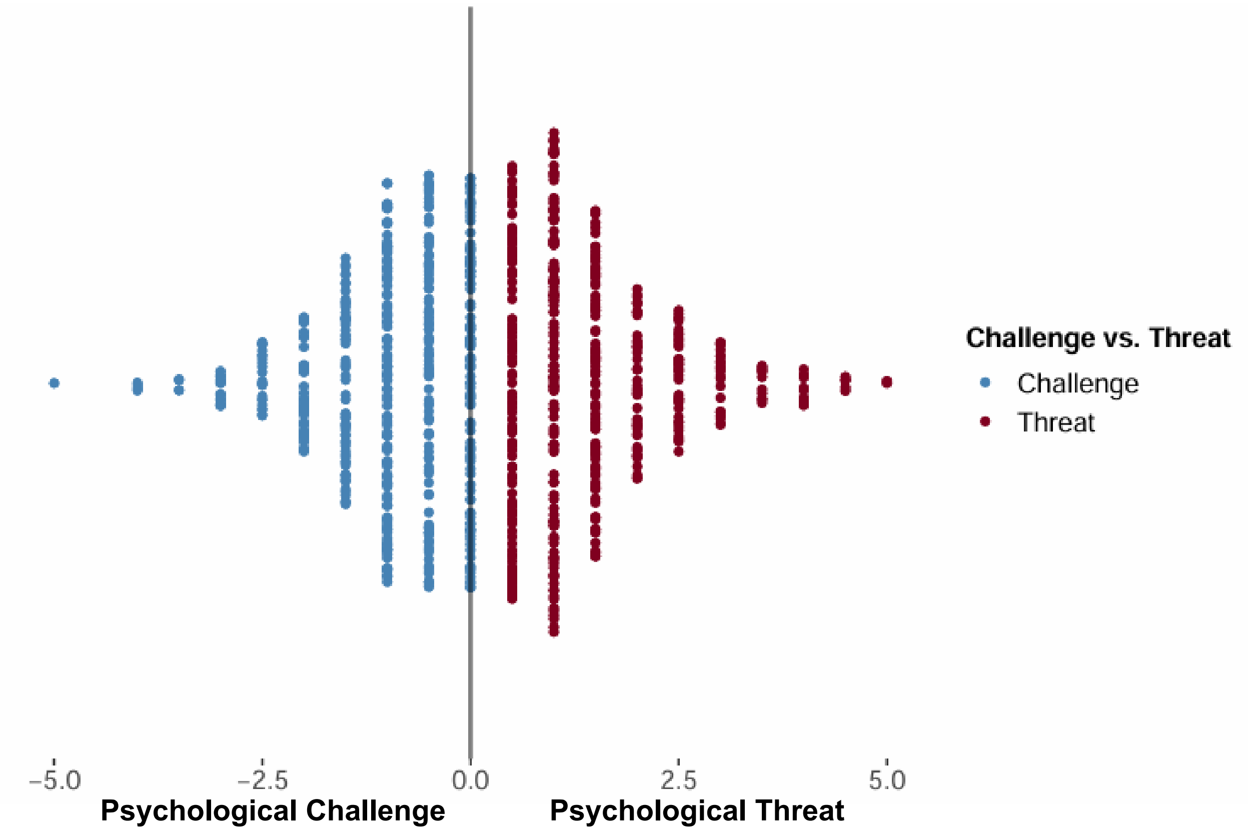

Threat Distribution Across Identity Groups

Just over half of students of color were threatened (54.02%) rather than challenged (45.98%; See Figure S4A). Among white students, about half were threatened (48.67%) and about half were challenged (51.32%). Students of color made up a larger portion of our study sample as compared to the larger population of physics students who completed the screening survey (see Table S8).

Among women and non-binary students, the majority were threatened (66.31%) rather than challenged (see Figure S4B; 33.69%). Among male students, the majority were challenged (59.97%) rather than threatened (40.03%). As a result, women and non-binary students made up a larger portion of our study sample as compared to the larger population of physics students who completed the screening survey (see Table S8).

Figure S4. Screening Survey Threat by Identification

A) Racial Identification

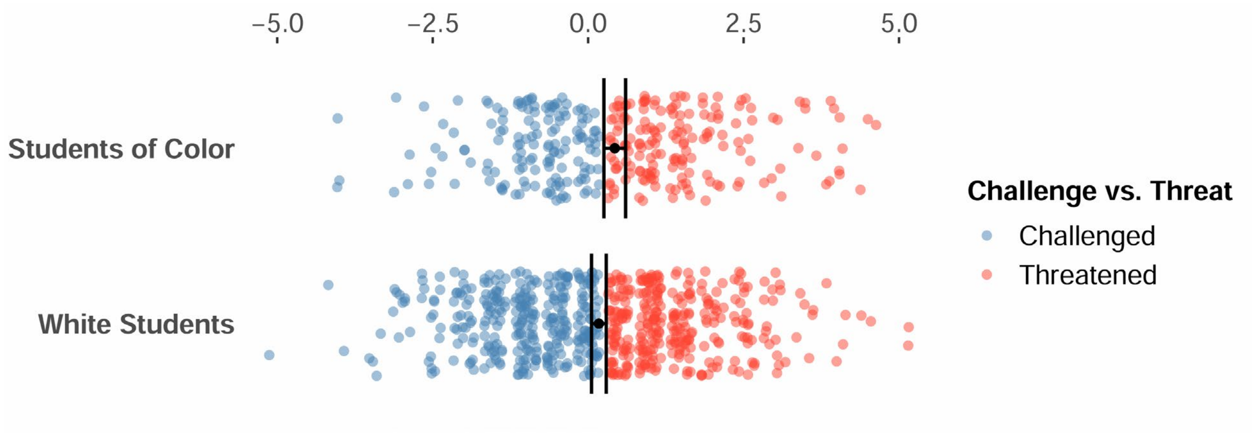

B) Gender Identification

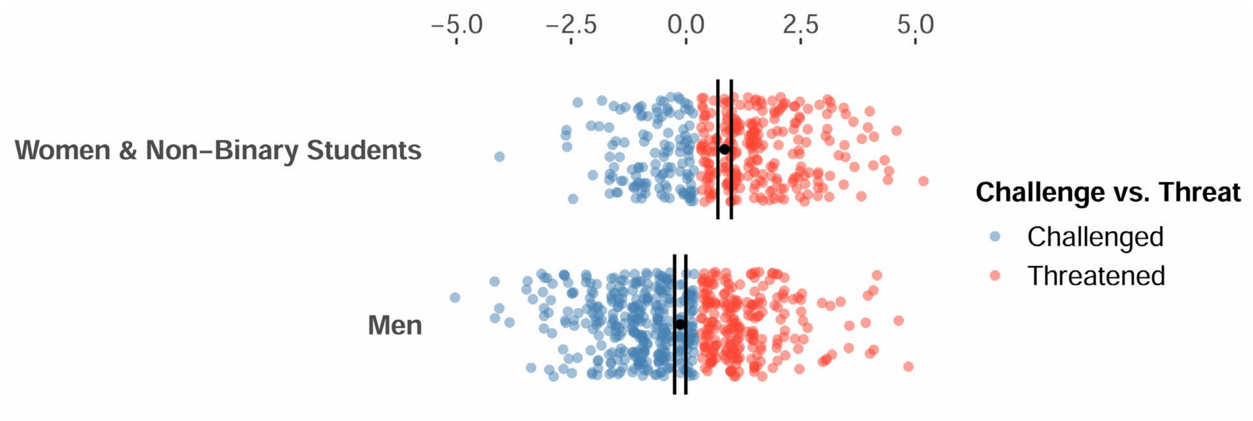

Table S8. Screening vs. Enrolled Participant Proportion by Demographic Category

	***Screening* (N = 954)**	***Enrolled* (N = 149)**
Analysis Classification		
Systemically Excluded Groups	57.97%	72.48%
Systemically Advantaged Group	42.03%	27.52%
Racial Identification		
Students of Color	32.08%	37.58%
White Students	67.92%	62.42%
Gender Identification		
Women & Non-Binary	39.52%	55.70%
Men	59.96%	44.30%

Note. Students of Color includes students who select multiple identities, one of which is not white. For this study, Middle Eastern/North African students are categorized as Students of Color and not White Students. Although this categorization is not universally agreed upon, it reflects how Middle Eastern/North African people are identified as not White, both by Middle Eastern/North African people and White people in the U.S. (2)
Systemically excluded groups includes all students of color (any gender), white women, and white non-binary people. Systemically Advantaged group includes white men.

Table S9. Descriptive Statistics for Psychological Threat across Demographic Category

	**N = 954**
Analysis Classification	
Systemically Excluded Groups	0.59 (1.52)
Systemically Advantaged Group	-0.20 (1.46)
Racial Identification	
Students of Color	0.43 (1.56)
White Students	0.17 (1.53)
Gender Identification	
Women & Non-Binary	0.84 (1.45)
Men	-0.13 (1.49)

Note. Table provides means and standard deviations in parentheses. Students of Color includes students who select multiple identities, one of which is not white. For this study, Middle Eastern/North African students are categorized as Students of Color and not White Students. Although this categorization is not universally agreed upon, it reflects how Middle Eastern/North African people are identified as not White, both by Middle Eastern/North African people and White people in the U.S. (2)
Systemically excluded groups includes all Students of Color (any gender), White women, and White non-binary people. Systemically advantaged group includes White men.

Figure S5. Threat Distribution by Racial & Gender Identification

A) **Women & Non-Binary Students (N = 377)**

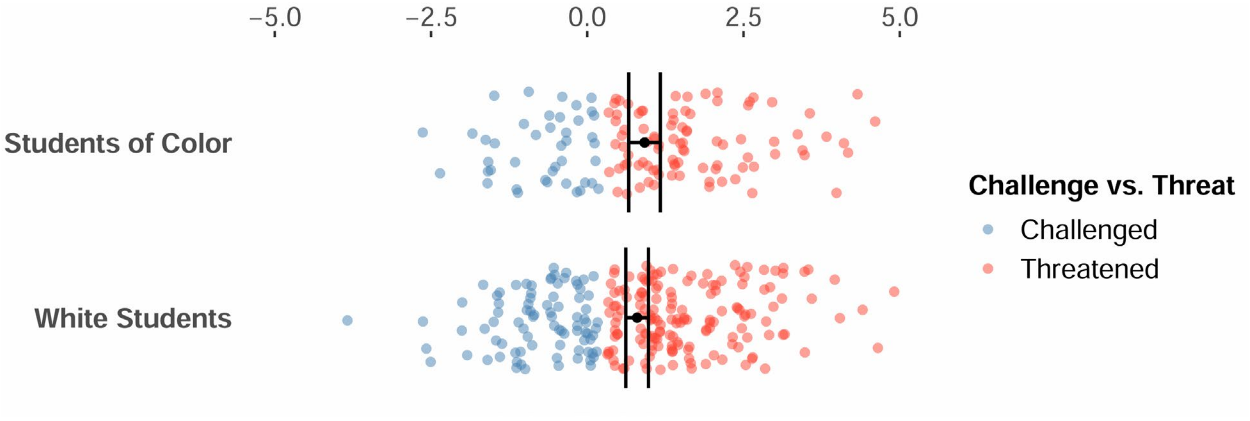

B) Male Students (N = 572)

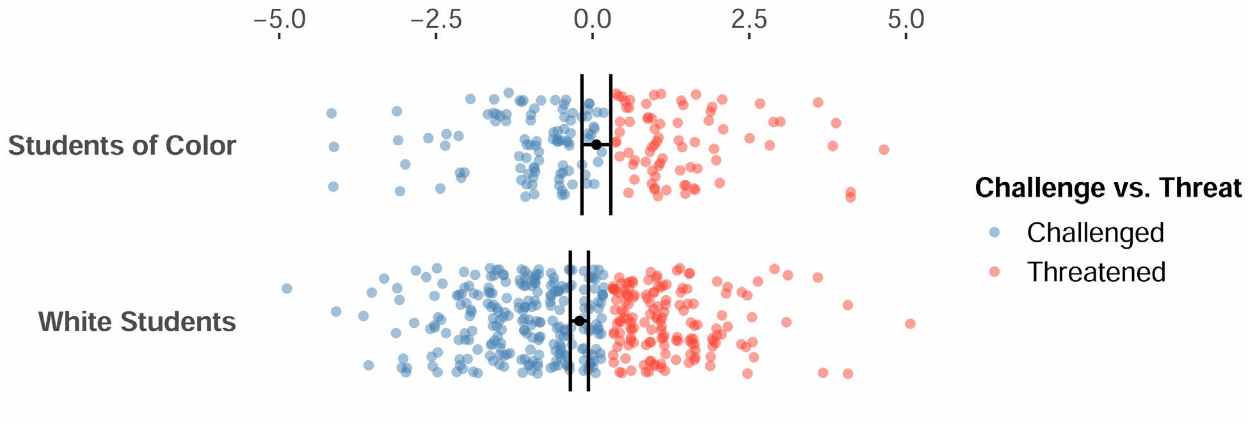

Enrolled Participants at Screening

Figure S6. Threat Distribution Across Identity Groups Among Enrolled Participants (N = 149)

A) **Analysis Classification**

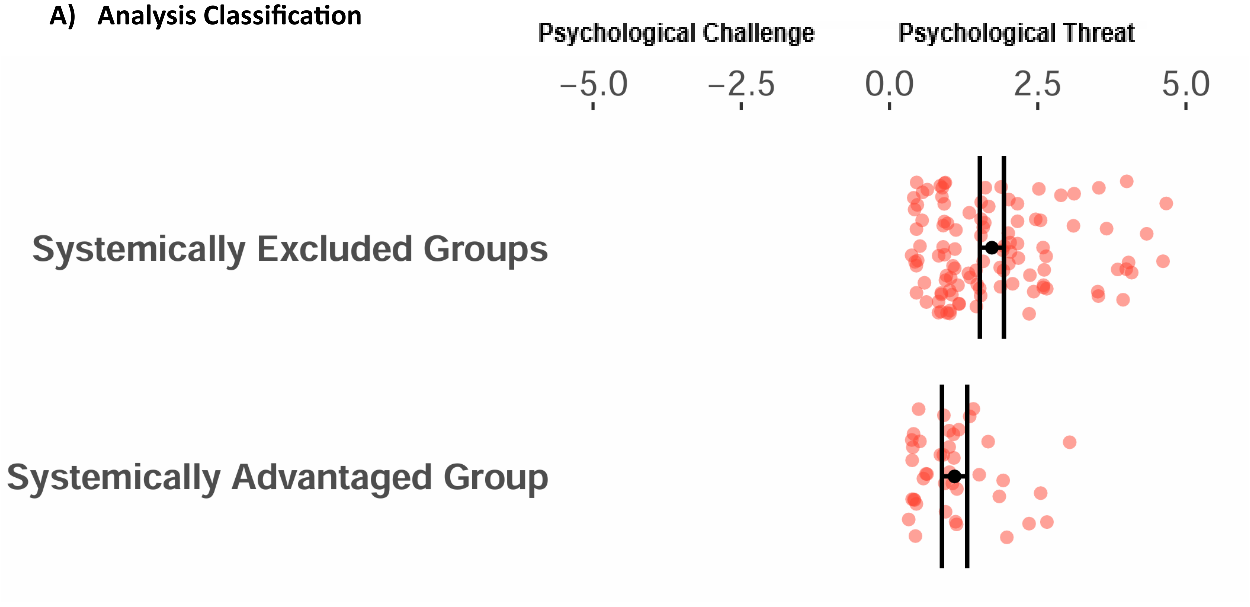

B) **Racial Identification**

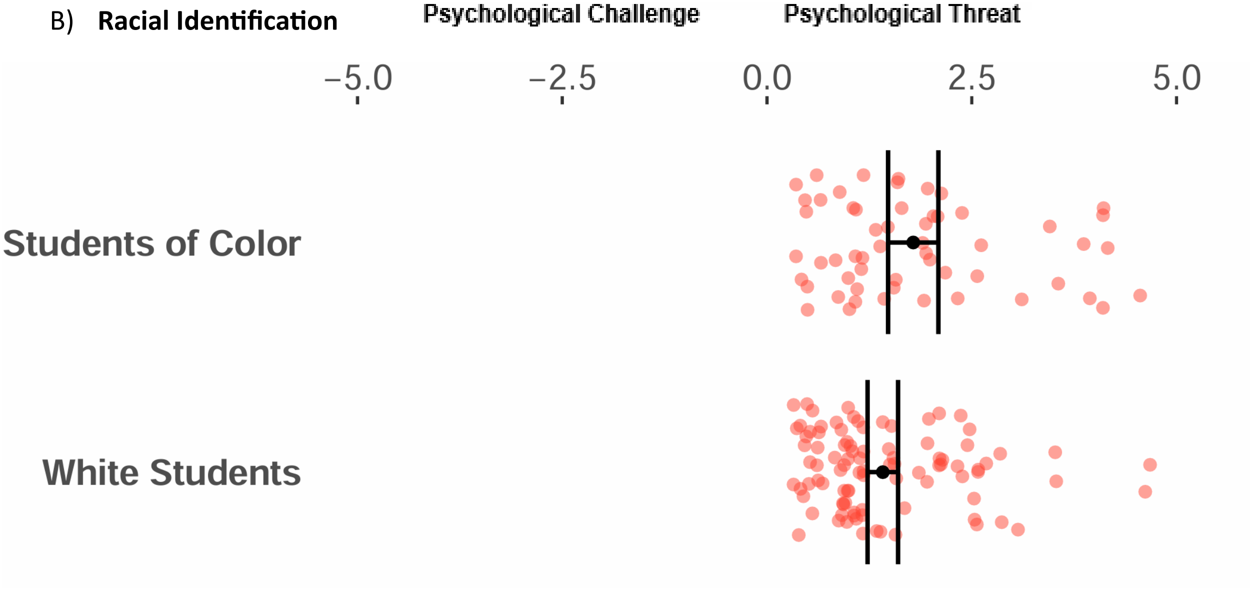

C) **Gender Identification**

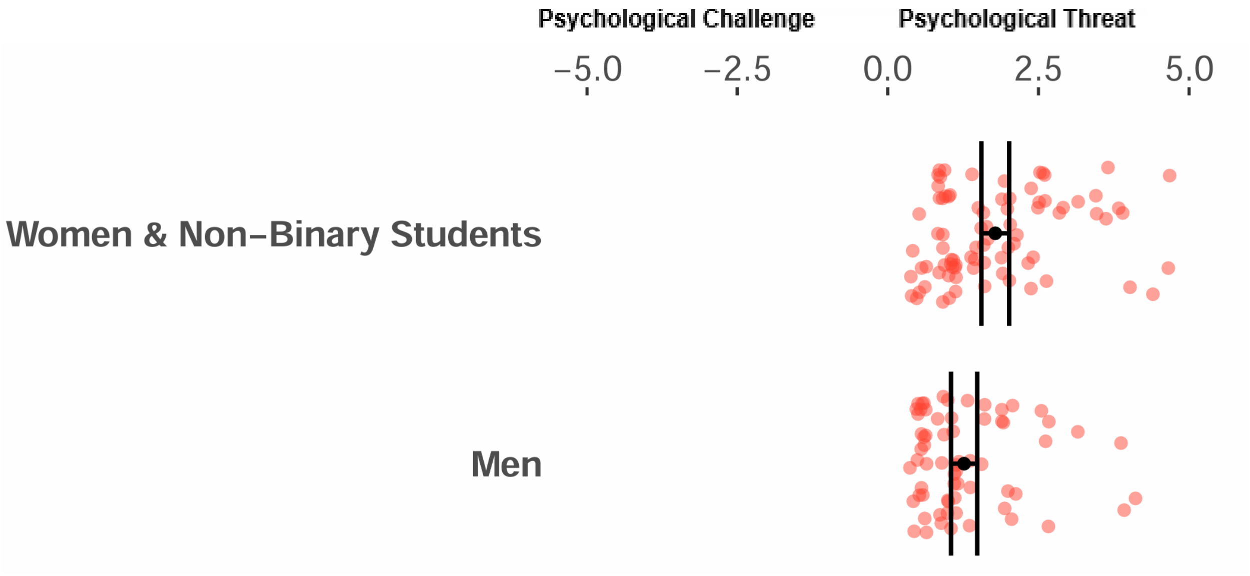

Not Enrolled and Ineligible Students

In the figures below, not enrolled refers to students who were screened as eligible but did not participate in the study.

Figure S7. Threat Distribution Across Identity Groups Among Not Enrolled Students (N = 279)

A) **Analysis Classification**

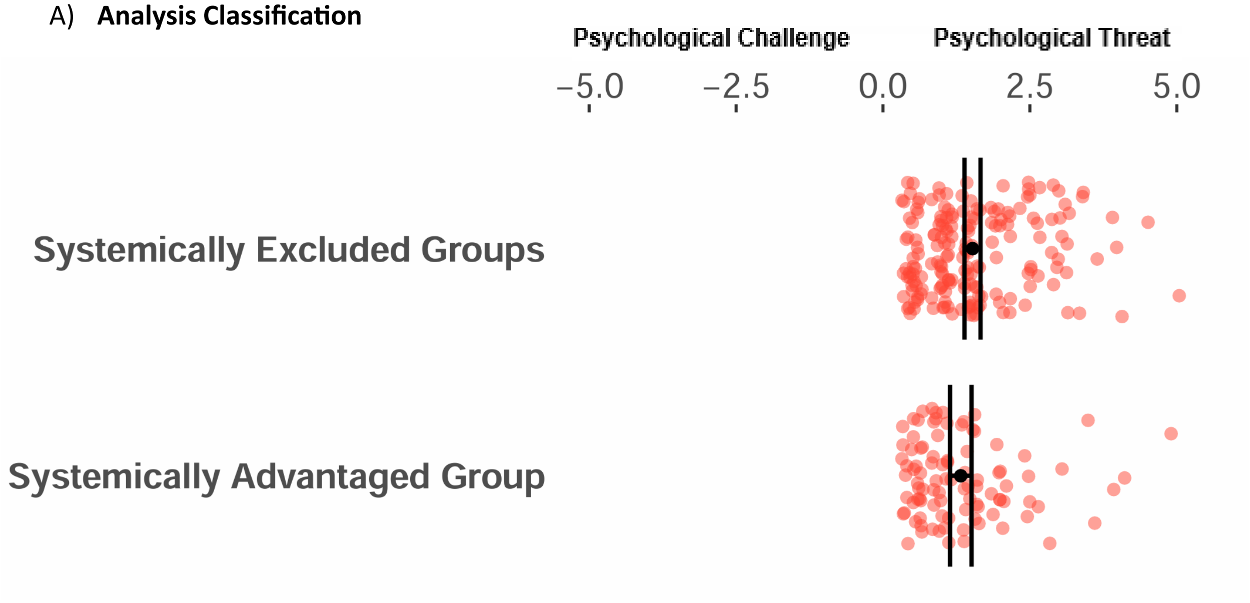

B) **Racial Identification**

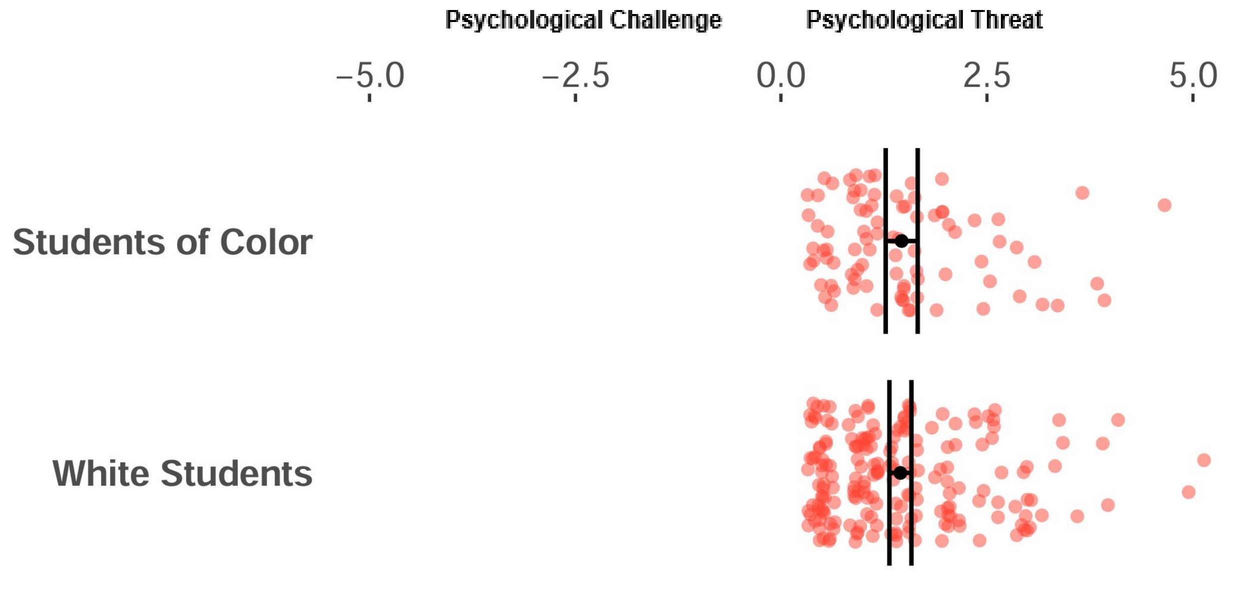

C) **Gender Identification**

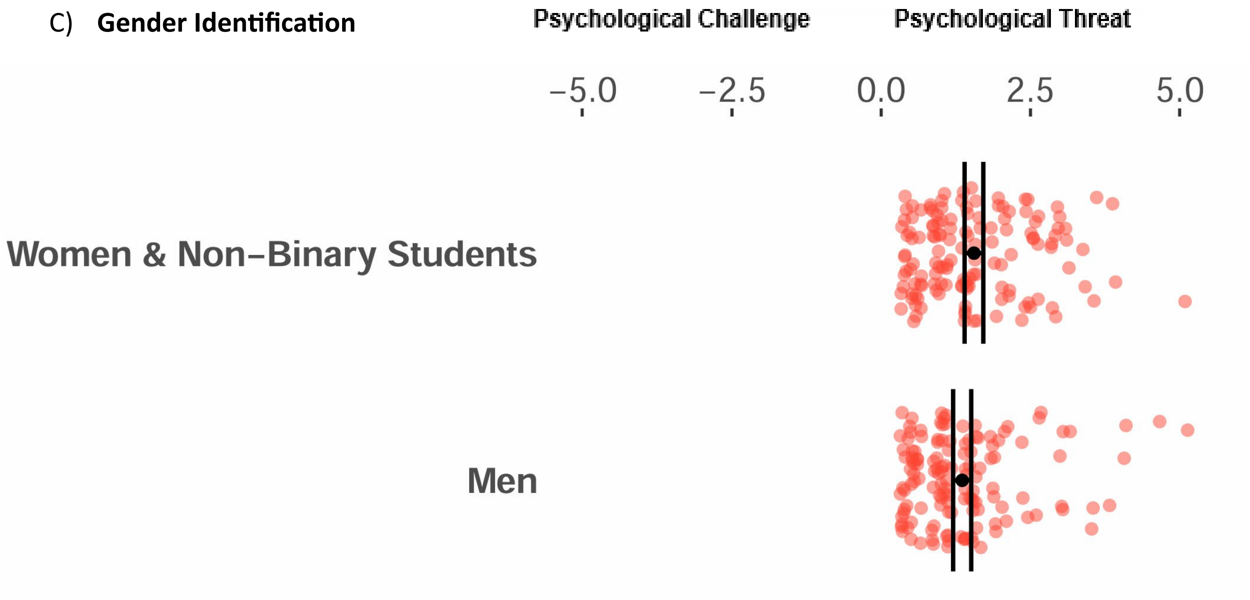

Figure S8. Threat Distribution Across Identity Groups Among Ineligible Students (N = 526)

A) **Analysis Classification**

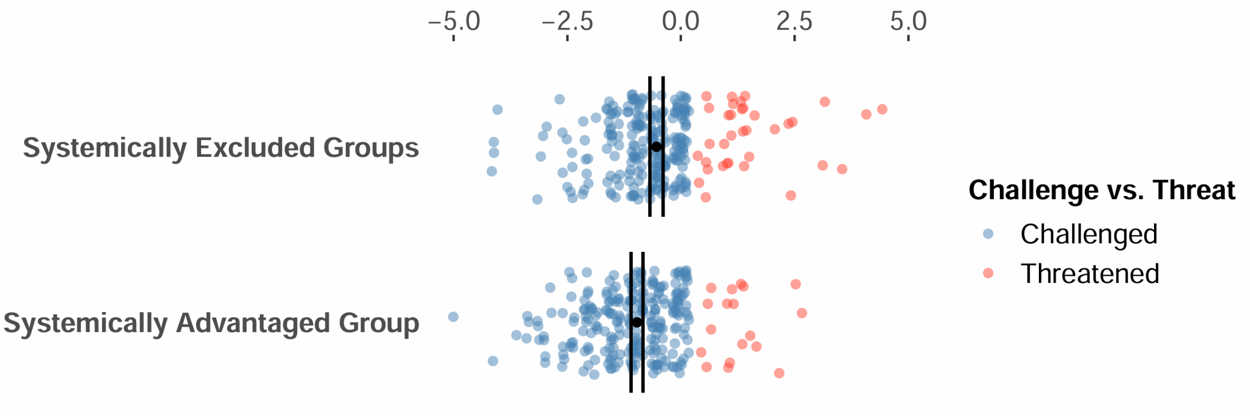

B) **Racial Identification**

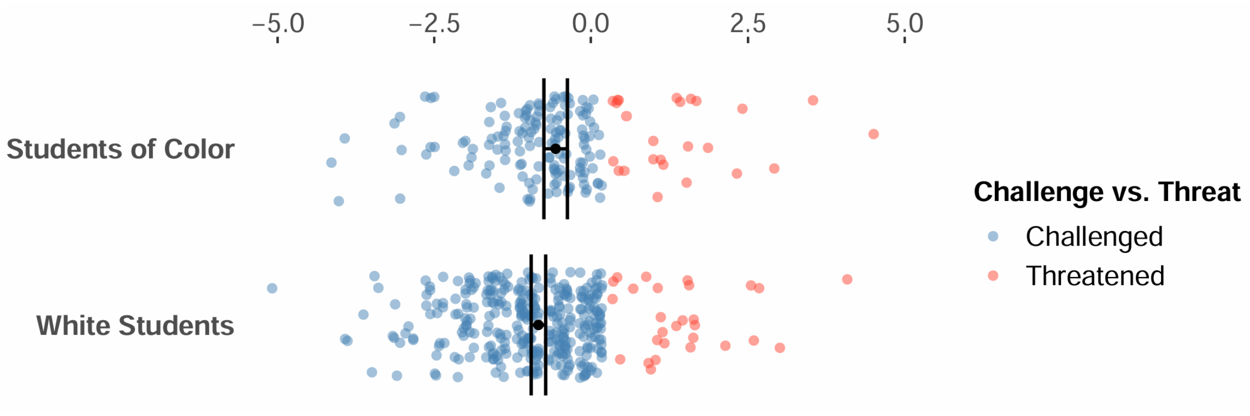

C) Gender Identification

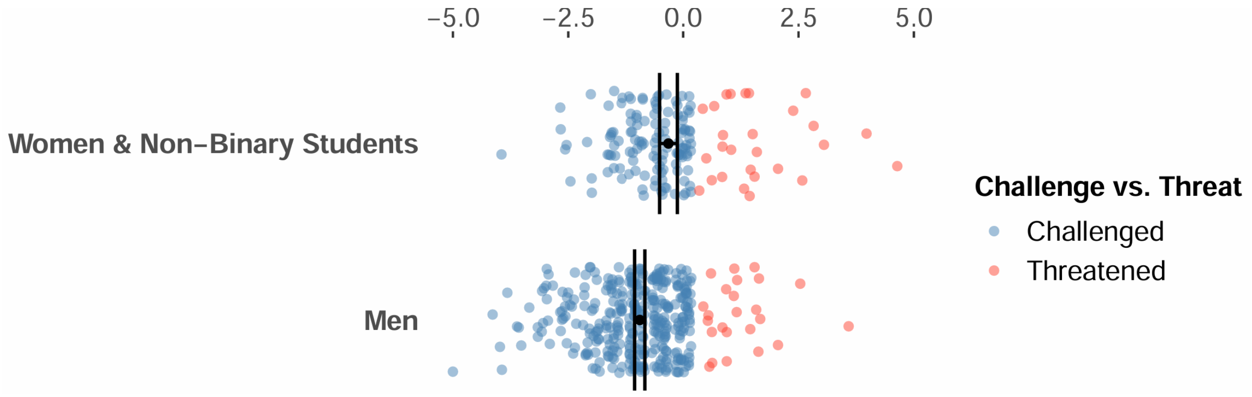

Note. Students could screen as threatened but still be ineligible for the experiment due to being younger than 18 years old or not English-speaking.

Table S10. Screening & Not Enrolled, Ineligible by Demographic Category

	Not Enrolled **(N = 279)**	***Ineligible*** **(N = 526)**
Analysis Classification		
Systemically Excluded Groups	65.59%	49.81%
Systemically Advantaged Group	34.41%	50.19%
Racial Identification		
Students of Color	31.18%	31.94%
White Students	68.82%	68.06%
Gender Identification		
Women & Non-Binary	50.18%	29.56%
Men	49.82%	70.44%

Note. Not Enrolled were students who screened as eligible but did not participate in the study. Ineligible were students who screened as ineligible because they were either challenged (not threatened), under 18, or not fluent in English.

Additional EMA Analyses

EMA Robustness Analyses – Without Covariates

A mixed linear model (MLM), with maximum likelihood estimation, tested condition (0 = no training control, 1 = mindfulness intervention) differences on momentary (EMA) psychological threat during the training week. Our model specified momentary psychological threat, resources or demands as the Level 1 outcome and modeled the Level 1 intercept as a random effect, using variance components covariance structure. We modeled Level 2 condition as a fixed effect.

As predicted, students who received the mindfulness intervention (vs. audiobook control) reported reduced momentary psychological threat [F (1, 146.04) = 7.47, p = .007, *pseudo* R^2 = .04]. Notably, while students in both conditions still experienced threat on average, it was significantly attenuated for those who received the mindfulness training.

We next examined whether mindfulness training affected momentary appraisals of resources, demands, or both. Students in the mindfulness training reported higher levels of coping resources than students in the control F (1, 145.94) = 9.90, p = .002, *pseudo* R^2 = .05]. However, conditions did not differ on demand appraisals [F (1, 146.21) = 1.75, p = .188, *pseudo* R^2 = .01].

EMA Moderation Analyses

Condition × Time

Mixed linear model (MLM), with maximum likelihood estimation, tested whether time moderated condition (0 = no training control, 1 = mindfulness intervention) differences on momentary (EMA) psychological threat, resources, or demands during the training week. Our models specified EMA threat, resources, or demands as the Level 1 outcome and modeled Level 1 time as a categorical variable (EMAs 1-6). As before, we modeled Level 1 intercept as a random effect, using variance components covariance structure, and Level 2 condition as a fixed effect.

Time did not moderate the effect of mindfulness training on psychological threat [Condition × Time Interaction: [*F* (5, 705.61) = 1.34, *p* = .245]. Additionally, time did not significantly moderate the effect on resources [Condition × Time Interaction: [*F* (5, 705.59) = 0.66, *p* = .65] or demands [Condition × Time Interaction: *F* (5, 706.05) = 1.76, *p* = .119].

Condition × Identity Classification

Mixed linear model (MLM), with maximum likelihood estimation, tested whether analysis classification (0 = systemically excluded groups, 1 = systemically advantaged group) moderated condition (0 = no training control, 1 = mindfulness intervention) differences on momentary (EMA) psychological threat, resources, or demands during the training week. Our model specified momentary psychological threat, resources, or demands as the Level 1 outcome and modeled the Level 1 intercept as a random effect, using variance components covariance structure. We modeled Level 2 condition and analysis classification as fixed effects.

Identification with systemically excluded or advantaged groups did not moderate the effect of mindfulness training on psychological threat [Condition × Analysis Classification Interaction: [*F* (1,133.83) = 0.00, *p* = .964]. Additionally, analysis classification did not significantly moderate the effect on resources [Condition × Analysis Classification: [*F* (1, 133.79) = 0.01, *p* = .932] or demands [*F* (1,133.81) = 0.00, *p* = .986].

Condition × Racial Identification

Mixed linear model (MLM), with maximum likelihood estimation, tested whether racial identification (0 = students of color, 1 = white students) moderated condition (0 = no training control, 1 = mindfulness intervention) differences on momentary (EMA) psychological threat, resources, or demands during the training week. Our model specified momentary psychological threat, resources, or demands as the Level 1 outcome and modeled the Level 1 intercept as a random effect, using variance components covariance structure. We modeled Level 2 condition and racial identification as fixed effects.

Racial identification did not moderate the effect of mindfulness training on psychological threat [Condition × Racial identification Interaction: [F (1,134.13) = 0.89, p = .346]. Additionally, racial identification did not significantly moderate the effect on resources [Condition × Racial Identification: [F (1, 134.09) = 0.22, p = .640] or demands [F (1, 134.22) = 1.40, p = .239].

Condition × Gender Identification

Mixed linear models (MLM), with maximum likelihood estimation, tested whether gender identification (0 = women and non-binary students, 1 = male students) moderated condition (0 = no training control, 1 = mindfulness intervention) differences on momentary (EMA) psychological threat, resources, or demands during the training week. Our model specified momentary psychological threat, resources, or demands as the Level 1 outcome and modeled the Level 1 intercept as a random effect, using variance components covariance structure. We modeled Level 2 condition and gender identification as fixed effects.

Gender identification moderated the effect of the training on EMA threat [F (1,133.99) = 4.11, p = .045]. Among women and non-binary students, mindfulness training significantly reduced momentary threat, t (134) = 3.15, p = .012. Men, however, did not show condition differences in momentary threat, t (134) = 0.09, p = .999. Gender identification did not significantly moderate the effect on resources [Condition × Gender Identification: F (1, 133.94) = 2.84 , p = .094] or demands [F (1,134.12) = 3.01, p = .085].

Readers will note that the main text talks about the absence of identity moderation, whereas these analyses indicate a significant Gender × Condition interaction on threat. We chose to interpret this interaction conservatively given that 1) the p-value for this interaction is close to the threshold of non-significance and 2) Gender × Condition interactions were not significant for either resources or demands. A significant interaction for the threat outcome alongside non-significant interactions for resources and demands (which are used to calculate threat) is difficult to interpret and could simply be the result of a

Type I error. Thus, we argue that the evidence in aggregate does not demonstrate consistent moderation of condition effects by gender identity.

Additional Longitudinal Threat Analyses

Mindfulness participants reported greater coping resources than control participants across posttest and 2-week follow-up assessments [Figure S9A; Condition x Time Interaction: *F* (3, 417.64) = 3.18, *p* = .024, *pseudo* R^2 =.08, 95% CI = [0.06, 0.22]. These findings are again consistent with a buffering effect whereby mindfulness mitigated longitudinal declines in coping resources.

Conditions did not differ in demand appraisals [*Figure S9B; F* (3, 417.14) = 2.01, *p* = .112, *pseudo* R^2 = .12, [0.09, 0.25]. Again, this null finding is consistent with prior research (3) and highlights a strength of the intervention approach – allowing instructors to maintain rigorous standards while still reducing students' psychological threat.

Figure S9. Long Term Effects of Mindfulness Training on Resource & Demand Appraisals

A) Resource Appraisals

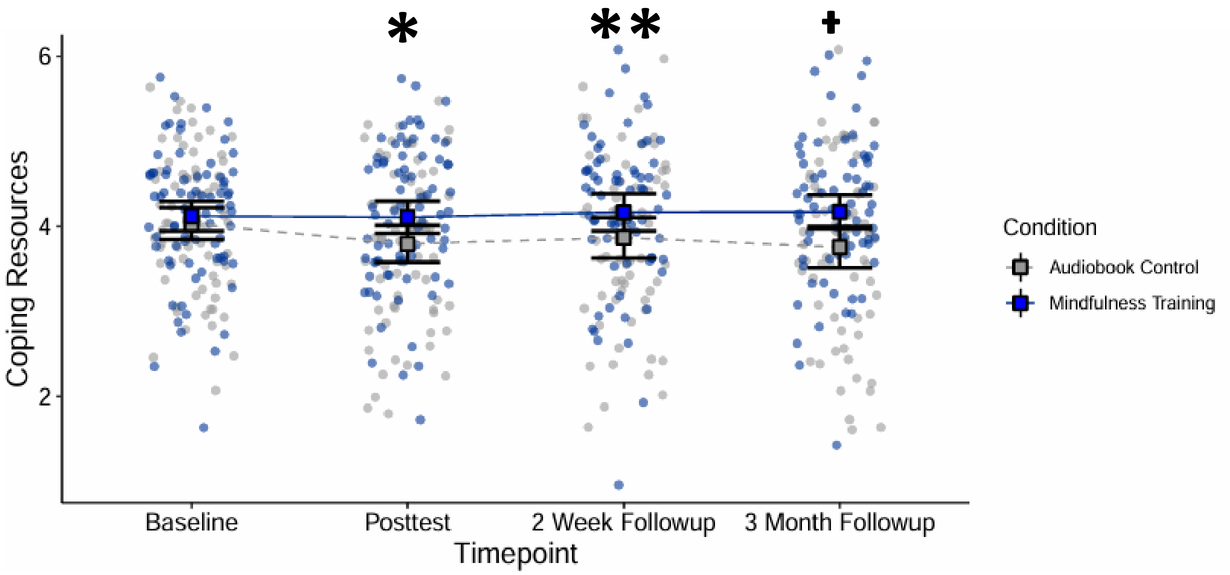

B) Demand Appraisals

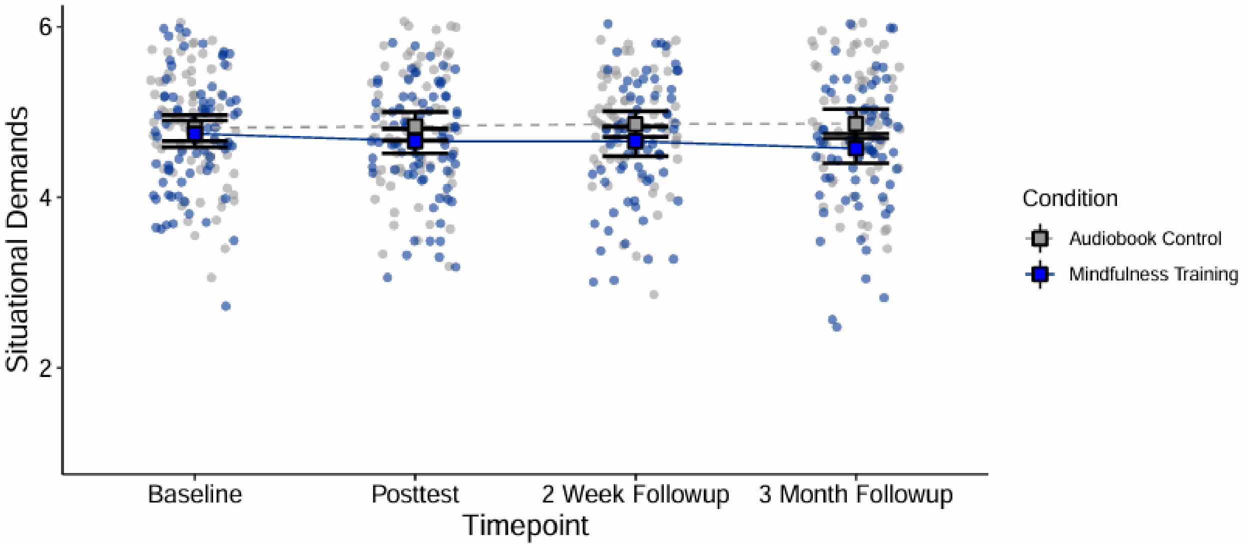

Note. Graphs present raw values. Error bars present 95% Confidence Interval

Table S11 provides details of the pairwise comparisons between mindfulness and audiobook control for threat and resources at each timepoint. Demands are not included below as the *Condition × Assessment* interaction was not significant.

Table S11. Pairwise Contrasts for Longitudinal Threat & Resources

	**Threat**	**Resources**
Baseline	t (147) = 0.74, p = .459	t (147) = -0.71, p = .480
Posttest	t (146) = 2.17, p = .031	t (146) = -2.18, p = .031
2 Week Follow-Up	t (138) = 2.17, p = .004	t (138) = -2.65, p = .009
3 Month Follow-Up	t (132) = 2.15, p = .033	t (132) = -1.84, p = .068

Longitudinal Robustness Analyses – without covariates

Mixed linear models, with maximum likelihood estimation, tested the longitudinal effects of condition (0 = audiobook control, 1 = mindfulness training) on threat, resources, or demands from baseline through posttest and follow-up surveys. Our model specified psychological threat as the Level 1 outcome and modeled Level 1 time as a categorical variable (baseline, posttest, 2-week follow-up, 3-month follow-up). As before, we modeled Level 1 intercept as a random effect, using variance components, and Level 2 condition as a fixed effect.

As predicted, students benefited from the mindfulness training relative to the audiobook control beyond the initial training week [Condition × Assessment Interaction: F (3, 417.55) = 3.98, p = .008, *pseudo* R^2 =.03]. Participants who received the mindfulness (vs. audiobook control) training reported lower psychological threat at posttest. This effect endured 2 weeks and 3 months later. Notably, while students in the audiobook condition experienced increased psychological threat over this period, the mindfulness training buffered students from this increase.

Like what was found with the EMA data, mindfulness participants reported greater coping resources than control participants, and this effect was observed at posttest, 2-week follow-up, and 3-month follow-up assessments [Condition x Time Interaction: F (3, 418.21) = 3.19, p = .024, *pseudo* R^2 =.03]. Again, conditions did not differ in demand appraisals in both studies [F (3, 417.14) = 1.95, p = .121, *pseudo* R^2 = .02].

Longitudinal Moderation Analyses

Condition × Time × Identity Classification

Mixed linear models, with maximum likelihood estimation, tested whether analysis classification (0 = systemically excluded groups, 1 = systemically advantaged group) moderated the longitudinal effects of condition (0 = audiobook control, 1 = mindfulness training) on threat from baseline through posttest and follow-up surveys. Our model specified psychological threat as the Level 1 outcome and modeled Level 1 time as a categorical variable (baseline, posttest, 2-week follow-up, 3-month follow-up). As before, we modeled Level 1 intercept as a random effect, using variance components, and Level 2 condition and analysis classification as fixed effects.

Analysis classification did not moderate the Condition × Assessment effect on psychological threat [3-Way Interaction: F (3, 411.24) = 0.21, p = .892]. Analysis classification also did not moderate the effect on resources [3-Way Interaction: [F (3, 411.60) = 0.07, p = .975] or demands [F (3, 411.21) = 0.68, p = .562].

Condition × Time × Racial Identification

Mixed linear models, with maximum likelihood estimation, tested whether racial identification (0 = students of color, 1 = white students) moderated the longitudinal effects of condition (0 = audiobook control, 1 = mindfulness training) on threat from baseline through posttest and follow-up surveys. Our model specified psychological threat as the Level 1 outcome and modeled Level 1 time as a categorical variable (baseline, posttest, 2-week follow-up, 3-month follow-up). As before, we modeled Level 1 intercept as a random effect, using variance components, and Level 2 condition and racial identification as fixed effects.

Racial identification did not moderate the Condition × Assessment effect on psychological threat [3-Way Interaction: F (3, 411.57) = 0.87, p = .459]. Racial identification also did not moderate the effect on resources [3-Way Interaction: [F (3, 411.99) = 0.87, p = .456] or demands [F (3, 411.59) = 1.28, p = .279].

Condition × Time × Gender Identification

Mixed linear models, with maximum likelihood estimation, tested whether gender identification (0 = women and non-binary students, 1 = male students) moderated the longitudinal effects of condition (0 = audiobook control, 1 = mindfulness training) on threat from baseline through posttest and follow-up surveys. Our model specified psychological threat as the Level 1 outcome and modeled Level 1 time as a categorical variable (baseline, posttest, 2-week follow-up, 3-month follow-up). As before, we modeled Level 1 intercept as a random effect, using variance components, and Level 2 condition and gender identification as fixed effects.

Gender identification did not moderate the Condition × Assessment effect on psychological threat [3-Way Interaction: F (3, 411.22) = 0.68, p = .563]. Gender identification also did not moderate the effect on resources [3-Way Interaction: [F (3, 411.65) = 0.40, p = .755] or demands F (3, 411.15) = 0.76, p = .519].

Additional Longitudinal Engagement Analyses

The main text presents MANCOVA findings for Cluster 1. Table S12 below reports the Condition × Assessment Interaction for the MANCOVAs and follow-up univariate tests for all 3 clusters.

Table S12. Condition × Assessment Effects on Engagement: Multivariate & Univariate

	**λ**	***F***	***df***	***p***	**η^2_p**
Cluster 1	**0.93**	**2.32**	**3, 387**	**.006**	**0.02**
Anxiety		4.76	3, 387	.003	0.04
Self-Efficacy		4.05	3, 387	.007	0.03
Belonging		2.49	3, 387	.060	0.02
Performance Avoidance Goals		0.98	3, 387	.402	< 0.01
Cluster 2	**0.94**	**1.61**	**3, 387**	**.064**	**0.01**
Physics Identity		3.46	3, 387	.017	0.03
Metacognitive Study Strategy Use		1.93	3, 387	.125	0.01
Effort		1.90	3, 387	.129	0.01
Failure Mindset		1.73	3, 387	.160	0.01
Intelligence Mindset		1.09	3, 387	.352	< 0.01
Cluster 3	**0.96**	**0.87**	**3, 387**	**.619**	**< 0.01**
Cognitive Study Strategy Use		2.21	3, 387	.087	0.02
Physics Value		1.10	3, 387	.349	< 0.01
Physics Interest		0.92	3, 387	.429	< 0.01
Mastery Approach Goals		0.61	3, 387	.607	< 0.01
Performance Approach Goals		0.52	3, 387	.669	< 0.01
Physics Help-Seeking Behavior		0.46	3, 387	.708	< 0.01

Note. All models control for self-reported gender (0 = women and non-binary students, 1 = men), semester week in which participant began the study, and semester of study enrollment.

Table S13. Cluster 1 Engagement Pairwise Comparisons

	**Baseline**	**Post-test**	**2-Week Follow-Up**	**3-Month Follow-Up**
**Anxiety**	t = 1.21, p = .229	t = 1.57, p = .118	t = 3.81, p < .001	t = 1.51, p = .132
**Self-Efficacy**	t = -1.05, p = .294	t = -2.16, p = .032	t = -3.40, p < .001	t = -2.52, p = .013
**Belonging**	t = -1.89, p = .060	t = -2.61, p = .009	t = -3.54, p = .001	t = -3.13, p = .002
**Performance Avoidance**	t = -1.35, p = .179	t = -0.24, p = .808	t = -1.14, p = .254	t = -0.31, p = .761

Note. Pairwise comparisons for Belonging and Performance Avoidance Goals are provided for transparency and completeness. Any significant pairwise comparisons for Belonging should be interpreted with caution since the univariate follow-up test did not reach traditional levels of significance (See Table S12).

Although the Condition × Assessment interaction was not significant for Cluster 2, we did follow up the significant univariate interaction for Identity. Figure S10 shows the fluctuation in physics identity at each assessment for both conditions. Table S14 provides the pairwise comparison statistics.

Figure S10. Effect of Condition on Physics Identity Across Time

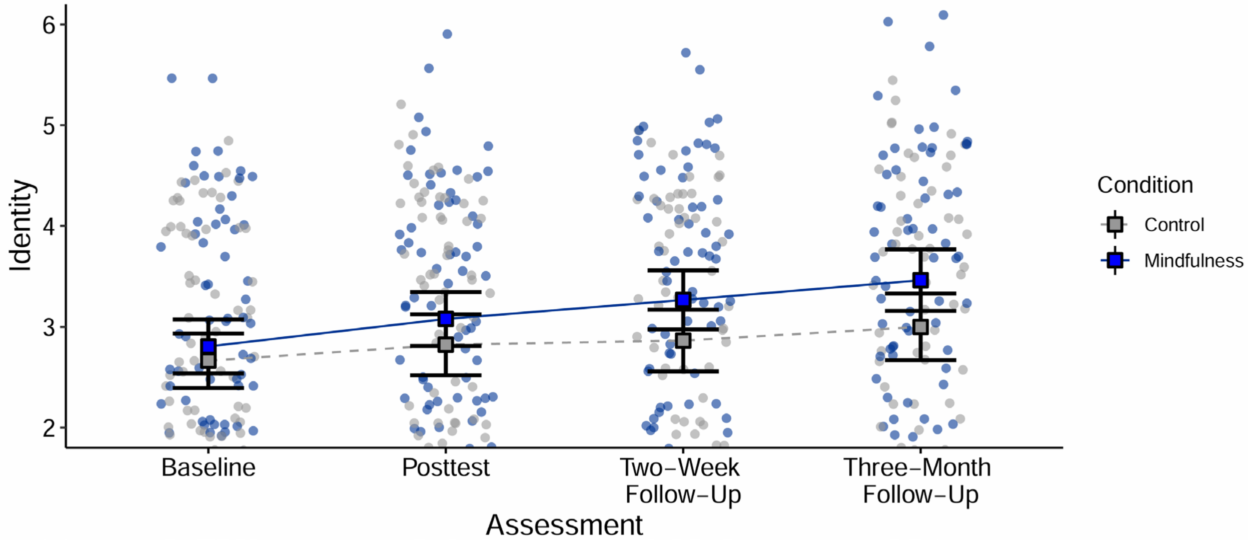

Note. Graphs present raw values. Error bars present 95% Confidence Intervals

Table S14. Pairwise Comparisons for Condition × Assessment Effect on Physics Identity

	t	*df*	*p*
**Baseline**	-0.42	149	.675
**Post-test**	-0.97	149	.332
**Two-Week Follow-Up**	-1.59	149	.115
**Three-Month Follow-Up**	-2.03	149	.044

Latent Threat Measurement Model Details

We chose to test psychological threat as a latent variable in the indirect effects analyses for both statistical and theoretical reasons. From a statistical standpoint, the latent variable approach provides a rigorous, precise test of the mediational pathway by leveraging the richness of the EMA data while limiting measurement error from biasing estimates of the mediator. Moreover, the latent variable approach allows us to directly model the relative contribution across each indicator, whereas a composite score approach would assume that all items contribute equally.

From a theoretical standpoint, psychological threat is treated as a state. As such, each measurement imperfectly reflects the state of threat, rather than directly representing the construct. Thus, the latent variable approach values the variability and sensitivity of each threat measurement, rather than treating each data point as an interchangeable snapshot in time.

To ensure our latent variable threat had good fit, we tested a measurement model. Our latent variable included 7 indicators of psychological threat (6 EMA surveys and the post-test survey). We included residual covariances for EMA surveys completed on the same day (e.g., Tuesday afternoon and Tuesday evening) as well as between the final EMA survey and posttest (given the relatively short time period between those two measurements). To account for missing data, we used full information maximum likelihood. The model indicated good fit across multiple indices: X^2 (10, N = 149) = 15.93, p = .102, *CFI* = .994, *RMSEA* = .063.

Additional Indirect-Effects Analyses

Although the Condition × Assessment interaction was not significant for Cluster 2, we conducted indirect effects analyses for identity at 2-Week Follow-Up (see Figure S12), given the significant univariate interaction for this outcome variable. Students in the mindfulness training condition (vs. audiobook control) had lower psychological threat during the training week and posttest, which, in turn, was associated with greater physics identity two weeks later

Figure S11. Indirect Effects of Psychological Threat

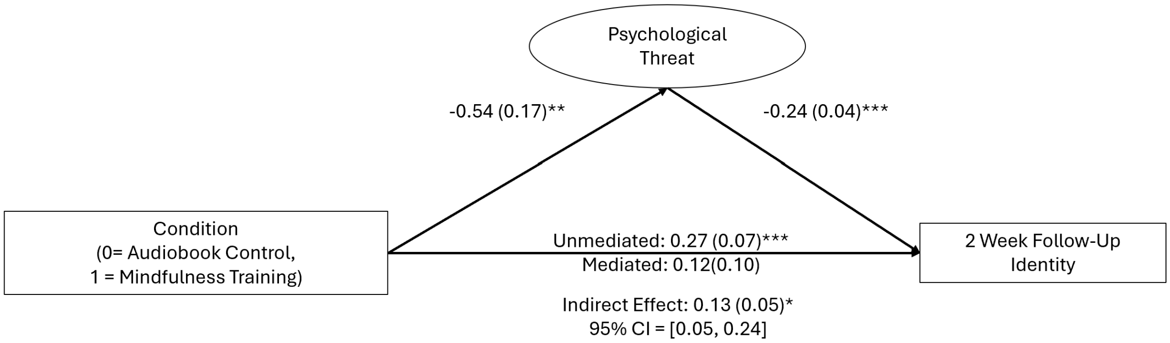

Finally, we tested indirect effect models for the same engagement variables (anxiety, self-efficacy, belonging, and identity) measured at 3-Month Follow-Up. Other than the change from 2-Week Follow-Up to 3-Month Follow-Up for each engagement outcome, mediation models for these tests were identical to the models for 2-Week Follow-Up (same latent variables and covariates).

Here, the indirect effects through psychological threat were significant for self-efficacy and physics identity at 3 months following the intervention (Figure S13).

Figure S12. 3-Month Follow-Up Self-Efficacy and Physics Identity Indirect Effect Models

A. Self-Efficacy

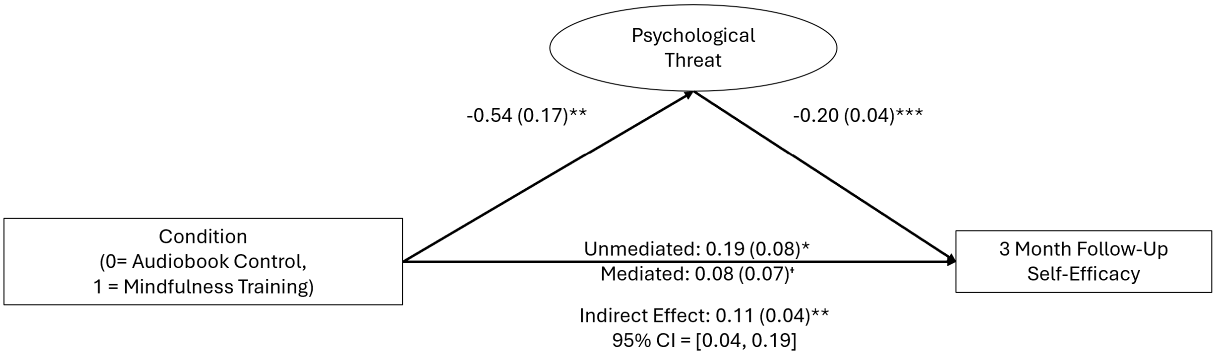

B. Physics Identity

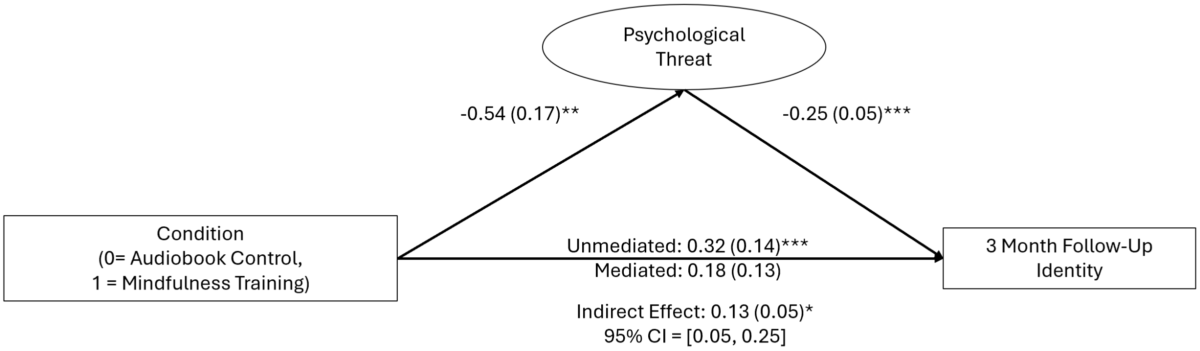

The indirect effects for anxiety and belonging were not significant but were in the predicted direction (Figure S14).

Figure S13. 3-Month Follow-Up Anxiety and Belonging Indirect Effect Models

A. Anxiety

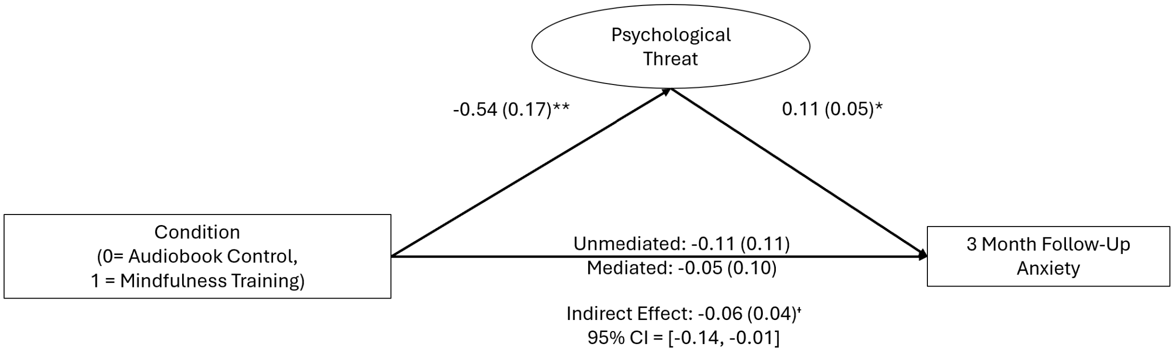

B. Belonging

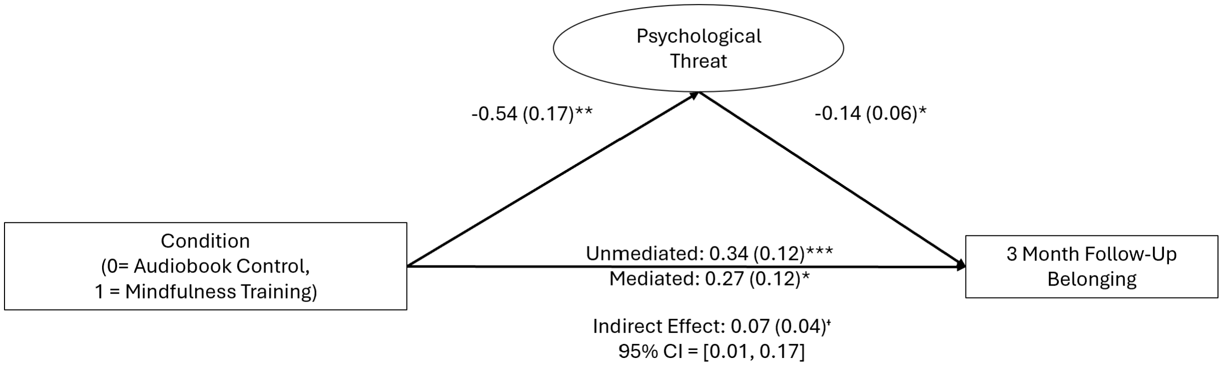

Pilot Study

The purpose of the pilot was to ensure the training was feasible and to serve as an initial test of its effect on psychological threat. The procedure for this study was nearly identical to that used in the main study. However, follow-up data for the pilot study were not collected at 3-Months (just 2-Weeks).

Screening Survey & Experiment Sample Demographic Information

Table S15. Pilot Sample Demographic Information (N = 27)

	N	% of sample
**Racial and Ethnic Identification** *(Participants could select more than one)*[a]		
Asian/Indian/Pacific Islander	6	22.22%
Black/African American	2	7.41%
Middle Eastern/North African	1	3.70%
White	19	70.37%
Students Who Selected Multiple Identities	1	3.70%
Asian/Indian/Pacific Islander, White		
**Racial and Ethnic Classification**		
Students of Color[b]	9	33.33%
White Students	18	66.67%
**Gender Identification**		
Women	11	40.74%
Men	16	59.26%
**Analysis Classification**		
Systemically Excluded Groups[c]	18	66.67%
Systemically Advantaged Group[d]	9	33.33%

Note. [a]*Frequencies for racial and ethnic identification sum to greater than 27 (100% of the sample) because participants were able to select multiple race and ethnicity categories.*

[b]*Students of Color includes students who select multiple identities, one of which is not white. For this study, Middle Eastern/North African students are categorized as Students of Color and not White Students. Although this categorization is not universally agreed upon, it reflects how Middle Eastern/North African people are identified as not White, both by Middle Eastern/North African people and White people in the U.S.* (2).

[c]*Systemically excluded group classification includes all students of color (any gender), white women, and white non-binary people*

[d]*Systemically advantaged group classification includes white men.*

Table S16. Pilot Screening Survey Demographic Information (N = 127)

	N	% of sample
**Racial and Ethnic Identification** *(Participants could select more than one):*[a]		
Asian/Indian/Pacific Islander	21	16.54%
Black/African American	6	4.72%
Hispanic/Latine	5	3.94%
Middle Eastern/North African	1	0.78%
White	101	79.50%
Students Who Selected Multiple Identities	6	4.72%
Asian/Indian/Pacific Islander, White		
Asian/Indian/Pacific Islander, White		
Black/African American, White		
Hispanic/Latine, White		
**Racial and Ethnic Classification**[b]		
Students of Color	32	25.20%
White Students	95	74.80%
**Gender Identification**		
Women	65	51.20%
Non-binary	1	0.78%
Men	61	48.03%
**Analysis Classification**		
Systemically Excluded Groups[c]	77	60.60%
Systemically Advantaged Group[d]	50	39.40%

Note. [a]*Frequencies for racial and ethnic identification provided categories and written responses sum to greater than 127 (100%) of the sample because participants were able to select multiple race and ethnicity categories.*
[b]*Students of Color includes students who select multiple identities, one of which is not white. For this study, Middle Eastern/North African students are categorized as Students of Color and not White Students. Although this categorization is not universally agreed upon, it reflects how Middle Eastern/North African people are identified as not White, both by Middle Eastern/North African people and White people in the U.S.* (2)
[c]*Systemically excluded groups includes all students of color (any gender), white women, and white non-binary people.*
[d]*Systemically advantaged group includes white men.*

Table S17. Pilot Study Baseline Equivalence

	Mindfulness	Control	Test	*df*	*p*	*95% CI*
**Group Size**	15	12				
**Demographics**						
Age	18.67 (0.62)	18.92 (1.31)	T	25	.552	[-0.63, 1.13]
Year in School	1	1	X^2	3	.417	N/A
Participant Gender			X^2	1	.806	N/A
Women & Non-Binary	9	7				
Men	6	5				
Racial/Ethnic Identification			X^2	1	.681	N/A
Students of Color	6	3				
White Students	9	9				
Analysis Classification			X^2	1	.999	N/A
Systemically Excluded Groups	10	8				
Systemically Advantaged Group	5	4				
**Psychological Threat**	0.34 (1.04)	0.90 (1.00)	T	25	.164	[-0.25, 1.38]
Demands	4.71 (0.72)	4.82 (0.43)	T	25	.633	[-0.36, 0.57]
Resources	4.37 (0.65)	3.92 (0.74)	T	25	.106	[-1.02, 0.11]

Note: Table provides means and standard deviations in parentheses. For Age, Threat, Demands, and Resources, we provide means with standard deviations in parentheses. For Year in School, we provide the modal response (1st year students). All other variables provide frequencies.

Pilot: Full Screening Survey Sample

Figure S14. Introductory Physics Challenge-Threat Distribution

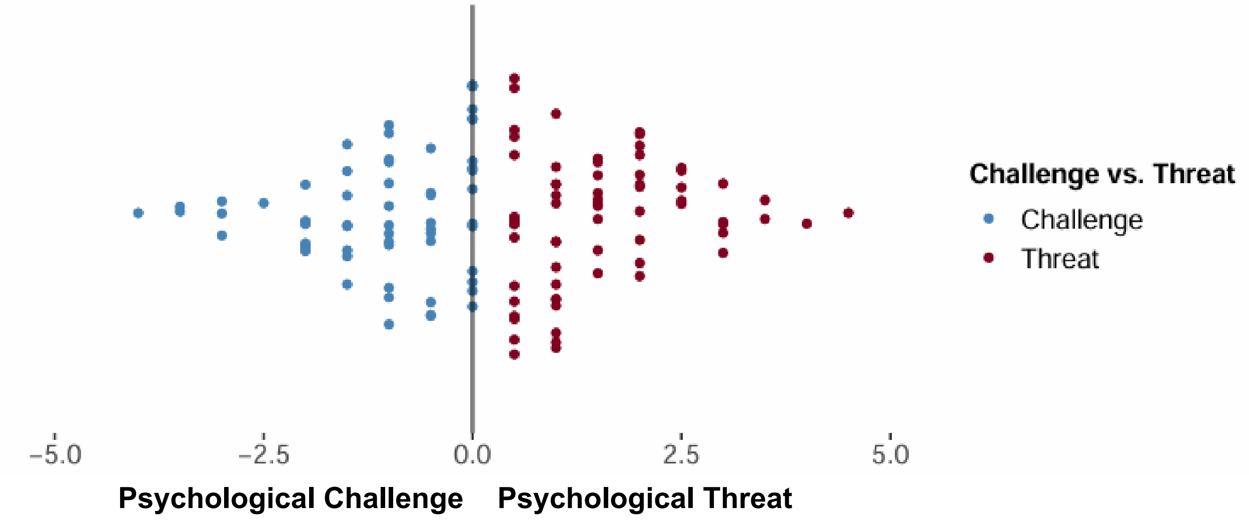

Pilot Study: Screening Survey Threat by Racial & Gender Identification

Half of the students of color (50.00%) were threatened and half (50.00%) were challenged. Among white students, the majority were threatened (53.70%) rather than challenged (46.30%). Students of color made up a larger portion of our pilot study sample as compared to the larger population of physics students who completed the screening survey (see Table S9).

Among women and non-binary students, the majority were threatened (66.67%) rather than challenged (see Figure S16; 33.33%). Among male students, the majority were challenged (62.30%) rather than threatened (37.70%). As a result, women and non-binary students made up a larger portion of our pilot study sample as compared to the larger population of physics students who completed the screening survey (see Table S18).

Figure S15. Screening Survey Threat by Identification (N = 127)

A) Racial Identification

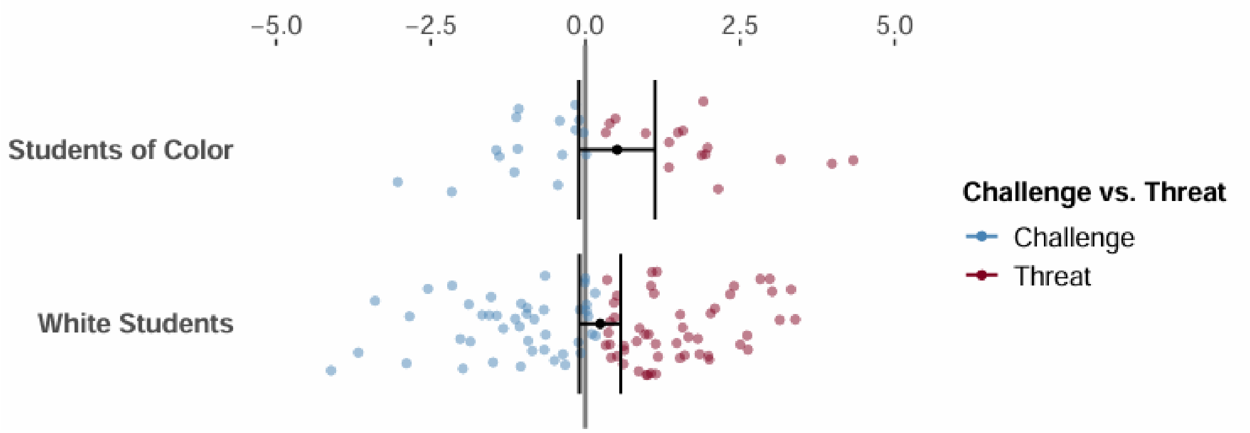

B) Gender Identification

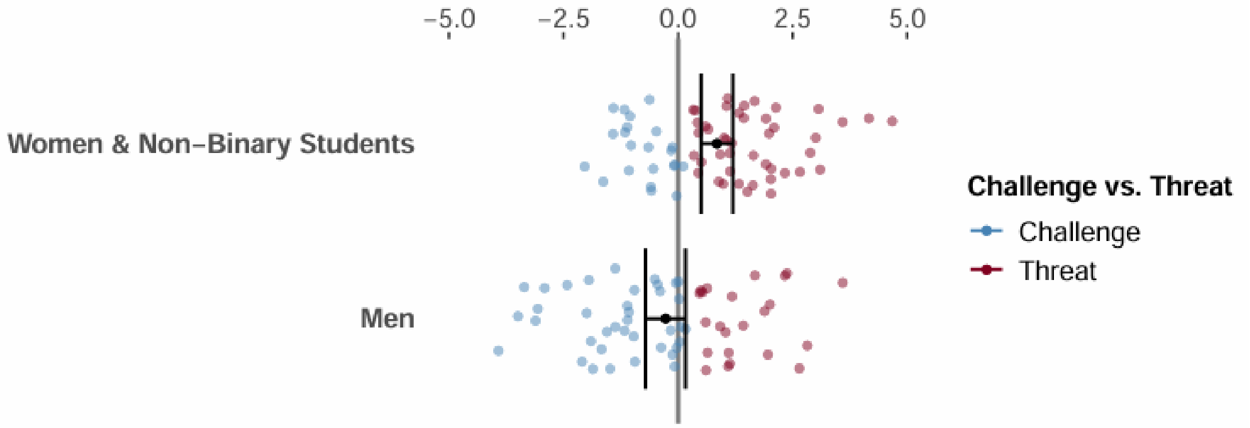

Table S18. Screening vs. Enrolled Participant Proportion by Demographic Category

	Pilot Study	
	Screening	***Enrolled***
Analysis Classification		
Systemically Excluded Groups	60.63%	66.67%
Systemically Advantaged Group	39.37%	33.33%
Racial Identification		
Students of Color	25.20%	33.33%
White Students	74.80%	66.67%
Gender Identification		
Women & Non-Binary	51.97%	59.26%
Men	48.03%	40.74%

Note. Students of Color includes students who select multiple identities, one of which is not white. For this study, Middle Eastern/North African students are categorized as Students of Color and not White Students. Although this categorization is not universally agreed upon, it reflects how Middle Eastern/North African people are identified as not White, both by Middle Eastern/North African people and White people in the U.S. (2)
Systemically excluded groups includes all students of color (any gender), white women, and white non-binary people. Systemically advantaged group includes white men.

Table S19. Descriptive Statistics for Psychological Threat across Demographic Category

	**N = 127**
Analysis Classification	
Systemically Excluded Groups	0.70 (1.48)
Systemically Advantaged Group	-0.06 (1.72)
Racial Identification	
Students of Color	0.52 (1.70)
White Students	0.24 (1.63)
Gender Identification	
Women & Non-Binary	0.85 (1.41)
Men	-0.27 (1.70)

Note. Table provides means and standard deviations in parentheses. Students of Color includes students who select multiple identities, one of which is not white. For this study, Middle Eastern/North African students are categorized as Students of Color and not White Students. Although this categorization is not universally agreed upon, it reflects how Middle Eastern/North African people are identified as not White, both by Middle Eastern/North African people and White people in the U.S. (2)

Systemically excluded groups includes all students of color (any gender), white women, and white non-binary people. Systemically advantaged group includes white men.

Figure S16. Threat Distribution by Racial and Gender Identification

A) **Women & Non-Binary Students (N = 66)**

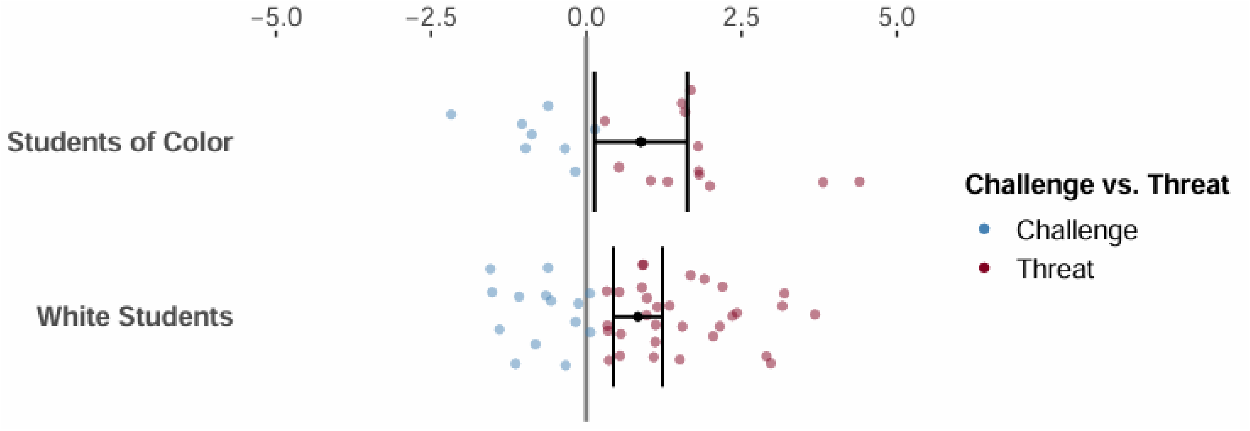

B) Male Students (N = 61)

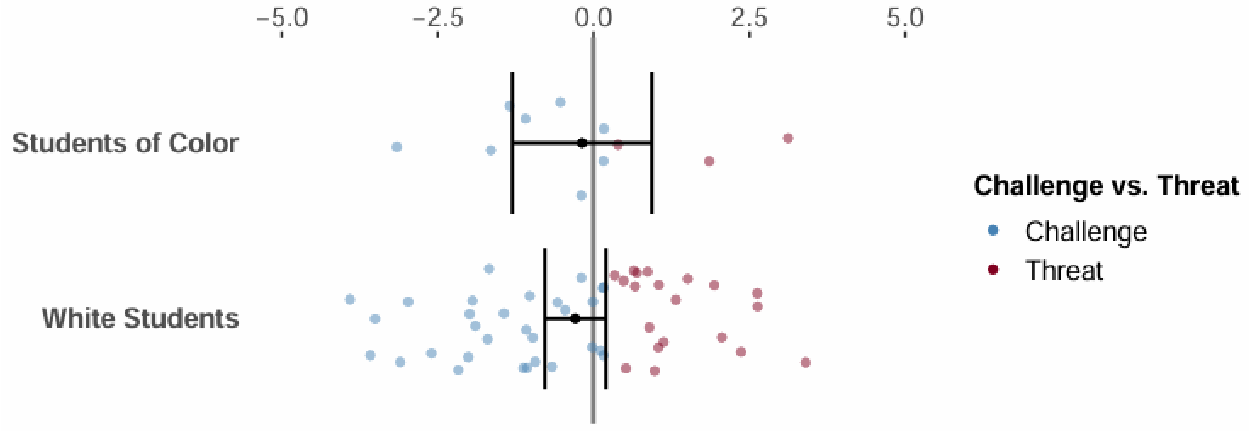

Enrolled Participants at Screening

Figure S17. Pilot Study Enrolled Participants (N = 27)

A) Analysis Classification

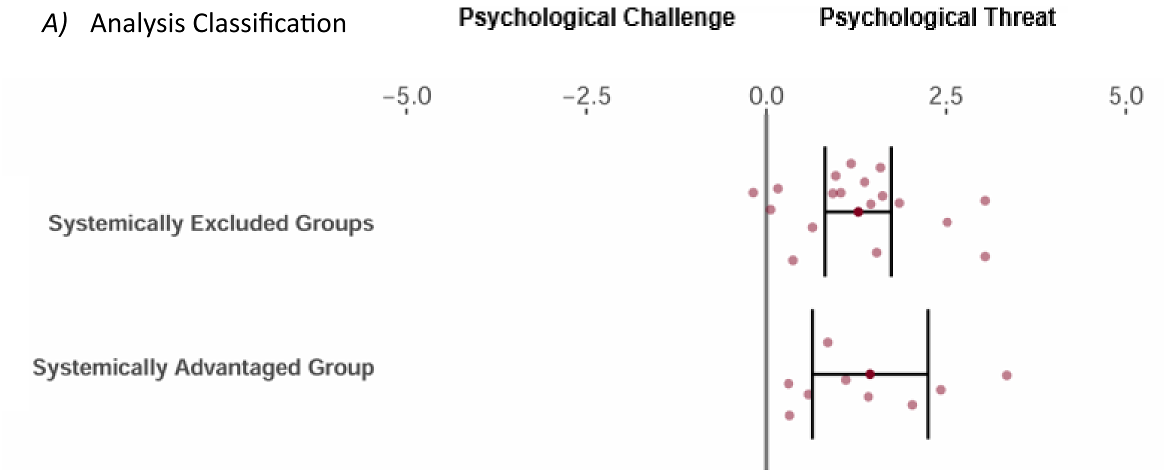

B) Racial Identification

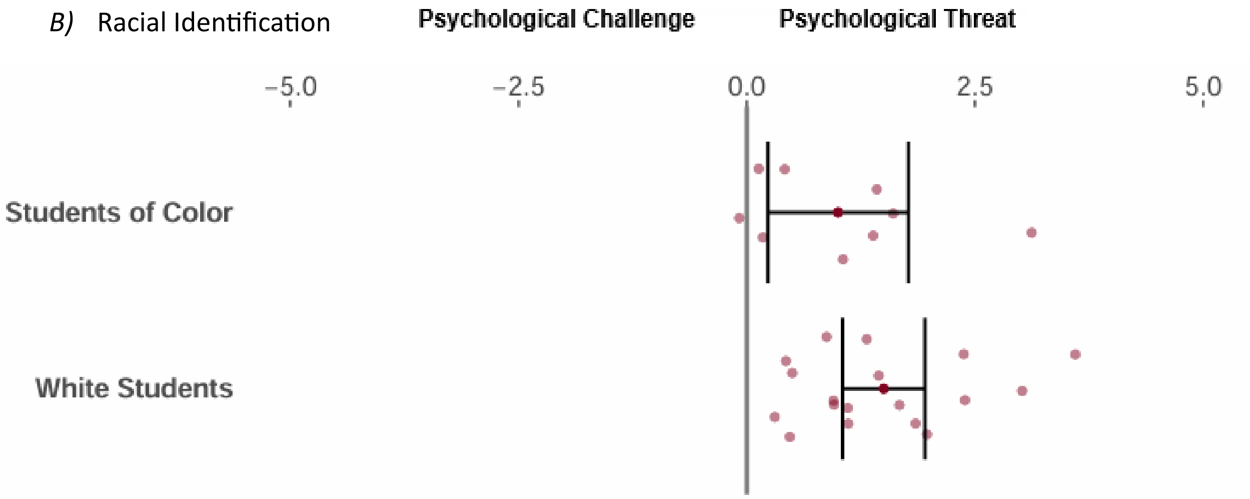

C) Gender Identification

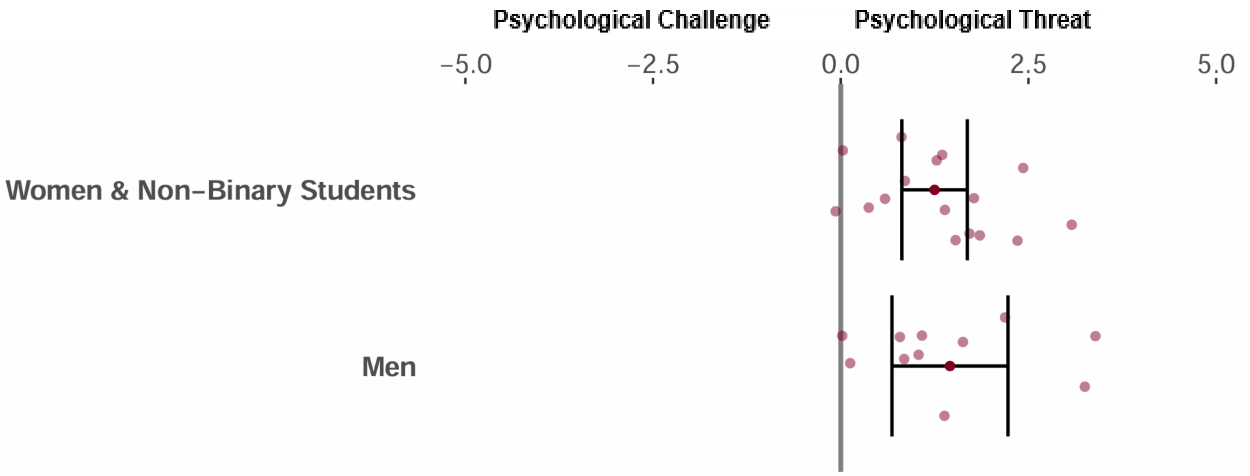

Not Enrolled and Ineligible Students

In the figures below, not enrolled refers to students who were screened as eligible but did not participate in the study.

Figure S18. Distribution of Threat Among Not Enrolled Students (N = 56)

A) **Analysis Classification**

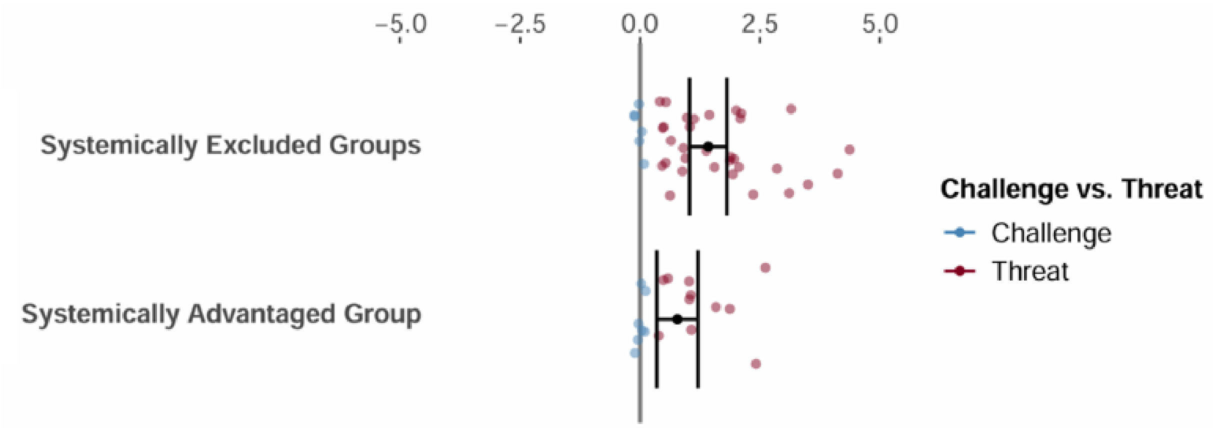

B) **Racial Identification**

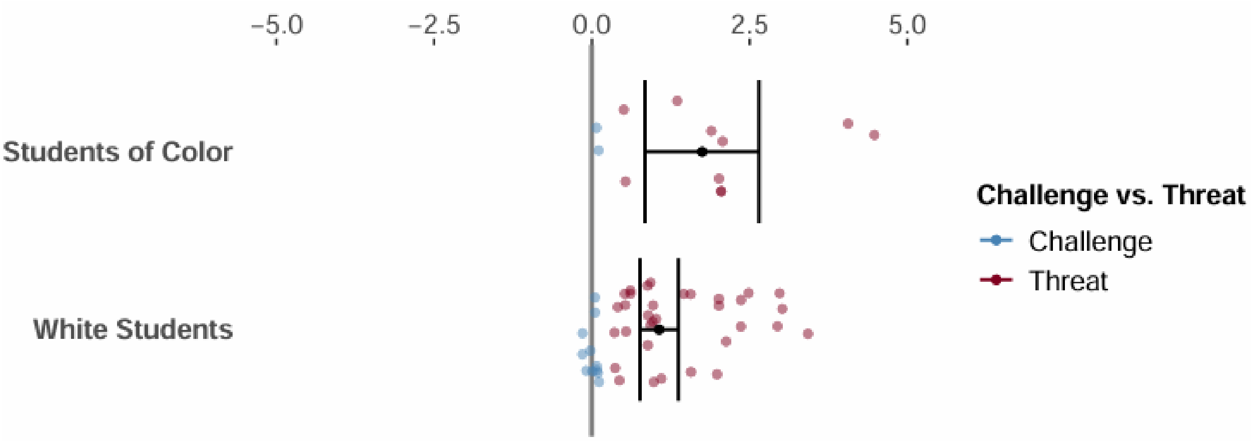

C) **Gender Identification**

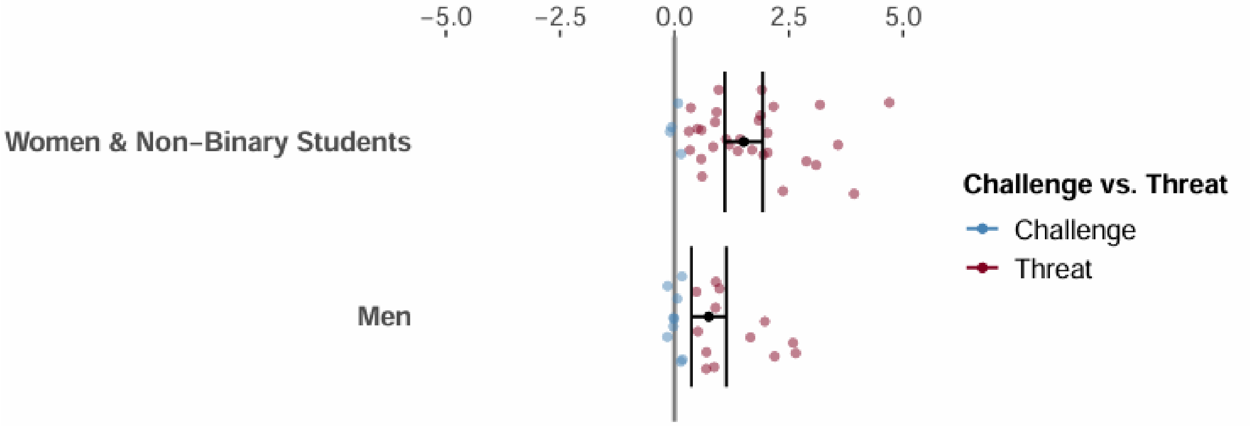

Figure S19. Threat Distribution Across Identity Groups Among Ineligible Students (N = 44)

A) **Analysis Classification**

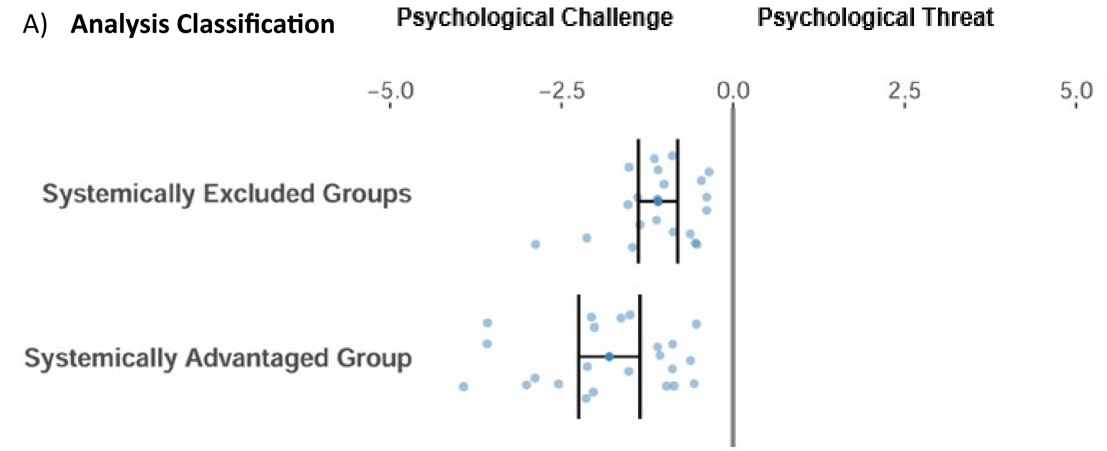

B) Racial Identification

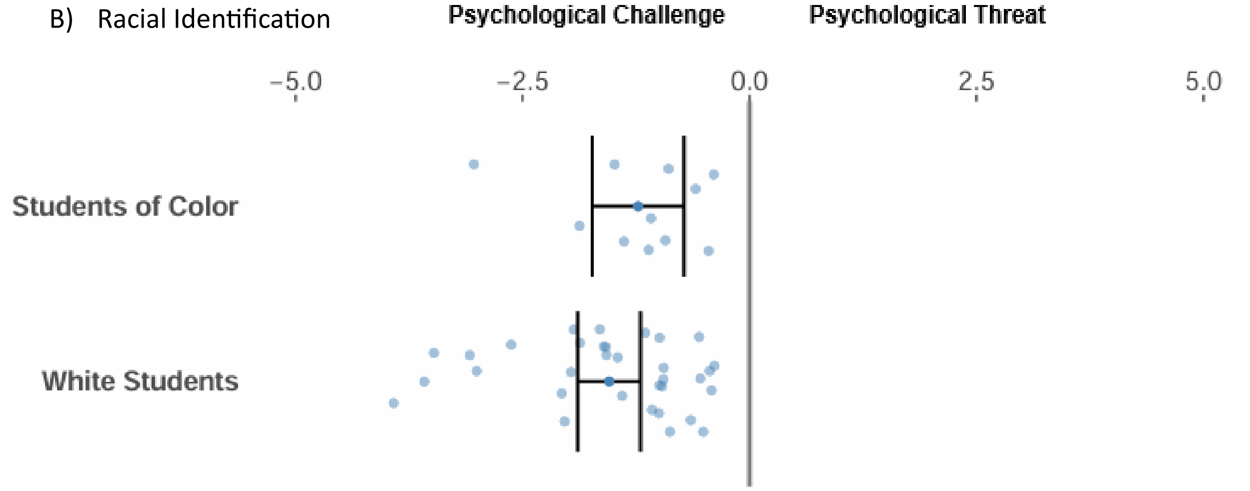

C) Gender Identification

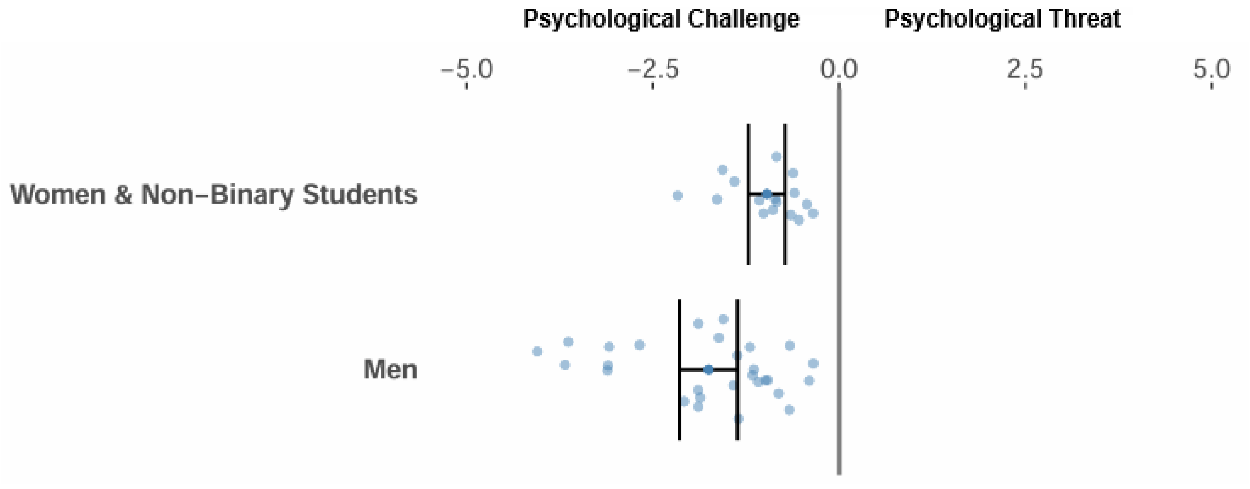

Table S20. Screening & Not Enrolled, Ineligible by Demographic Category

	Not Enrolled	***Ineligible***
Analysis Classification		
Systemically Excluded Groups	67.86%	47.73%
Systemically Advantaged Group	32.14%	52.27%
Racial Identification		
Students of Color	21.43%	25.00%
White Students	78.57%	75.00%
Gender Identification		
Women & Non-Binary	60.71%	36.36%
Men	39.29%	63.36 %

Note. Not Enrolled were students who screened as eligible but did not participate in the study. Ineligible were students who screened as ineligible because they were either challenged (not threatened), under 18, or not fluent in English.

Pilot EMA Analyses

Momentary Threat, Resources, & Demands

As predicted, students who received the mindfulness intervention (vs. audiobook control) reported reduced momentary psychological threat [*F* (1, 24.00) = 7.72, *p* = .010, *pseudo* R^2 = .23, 95% CI = [0.06, 0.49]. Students in the mindfulness training reported higher levels of coping resources than students in the control [*F* (1, 24.02) = 6.79, *p* = .016, *pseudo* R^2 = .20, 95% CI = [0.05, 0.46]. However, conditions did not differ on demand appraisals [*F* (1, 23.97) = 6.79, *p* = .069, *pseudo* R^2 = .12, 95% CI = [0.01, 0.37].

EMA Robustness Analyses – Without Covariates

A mixed linear model (MLM), with maximum likelihood estimation, tested condition (0 = no training control, 1 = mindfulness intervention) differences on momentary (EMA) psychological threat during the training week. Our model specified momentary psychological threat, resources or demands as the Level 1 outcome and modeled the Level 1 intercept as a random effect, using variance components covariance structure. We modeled Level 2 condition as a fixed effect.

As predicted, students who received the mindfulness intervention (vs. audiobook control) reported reduced momentary psychological threat [*F* (1, 25.01) = 7.09, *p* = .013, *pseudo* R^2 = .17]. Notably, while students in both conditions still experienced threat on average, it was significantly attenuated for those who received the mindfulness training.

We next examined whether mindfulness training affected momentary appraisals of resources, demands, or both. Students in the mindfulness training reported higher levels of coping resources than students in the control [*F* (1, 25.02) = 6.32, *p* = .019, *pseudo* R^2 = .15]. However, conditions did not differ on demand appraisals [*F* (1, 24.99) = 3.50, *p* = .073, *pseudo* R^2 = .09].

EMA Moderation Analyses

Condition × Time

Mixed linear model (MLM), with maximum likelihood estimation, tested whether time moderated condition (0 = no training control, 1 = mindfulness intervention) differences on momentary (EMA) psychological threat, resources, or demands during the training week. Our models specified EMA threat, resources, or demands as the Level 1 outcome and modeled Level 1 time as a categorical variable (EMAs 1-6). As before, we modeled Level 1 intercept as a random effect, using variance components covariance structure, and Level 2 condition as a fixed effect.

Time did not moderate the effect of mindfulness training on psychological threat [Condition × Time Interaction: [F (5, 124.017) = 0.78, p = .567]. Additionally, time did not significantly moderate the effect on resources [Condition × Time Interaction: [F (5, 124.017) = 0.81, p = .545] or demands [Condition × Time Interaction: F (5, 124.00) = 0.60, p = .70].

Condition × Analysis Classification

Mixed linear model (MLM), with maximum likelihood estimation, tested whether analysis classification (0 = systemically excluded groups, 1 = systemically advantaged group) moderated condition (0 = no training control, 1 = mindfulness intervention) differences on momentary (EMA) psychological threat, resources, or demands during the training week. Our model specified momentary psychological threat, resources, or demands as the Level 1 outcome and modeled the Level 1 intercept as a random effect, using variance components covariance structure. We modeled Level 2 condition and analysis classification as fixed effects.

Identification with systemically excluded or advantaged groups did not moderate the effect of mindfulness training on psychological threat [Condition × Analysis Classification Interaction: [F (1,22.99) = 1.41, p = .247]. Additionally, analysis classification did not significantly moderate the effect on resources [Condition × Analysis Classification: [F (1,23.01) = 0.34, p = .565] or demands [F (1, 22.96) = 2.06, p = .165].

Condition × Racial Identification

Mixed linear model (MLM), with maximum likelihood estimation, tested whether racial identification (0 = students of color, 1 = white students) moderated condition (0 = no training control, 1 = mindfulness intervention) differences on momentary (EMA) psychological threat, resources, or demands during the training week. Our model specified momentary psychological threat, resources, or demands as the Level 1 outcome and modeled the Level 1 intercept as a random effect, using variance components covariance structure. We modeled Level 2 condition and racial identification as fixed effects.

Racial identification did not moderate the effect of mindfulness training on psychological threat [Condition × Racial identification Interaction: [F (1,23.01) = 3.02, p = .096]. Additionally, racial identification did not significantly moderate the effect on resources [Condition × Racial Identification: [F (1,23.01) = 0.64, p = .43]. The effect of condition on demands differed by racial identification [F (1, 23.00) = 5.10, p = .034]. Mindfulness training (vs. audiobook control) reduced perceived demands for white students [t (23) = 3.16, p = .027]. Among students of color, conditions did not differ in perceived demands [t (23) = -0.61, p = .999].

Condition × Gender Identification

Mixed linear models (MLM), with maximum likelihood estimation, tested whether gender identification (0 = women and non-binary students, 1 = male students) moderated condition (0 = no training control, 1 = mindfulness intervention) differences on momentary (EMA) psychological threat, resources, or demands during the training week. Our model specified momentary psychological threat, resources, or demands as the Level 1 outcome and modeled the Level 1 intercept as a random effect, using variance components covariance structure. We modeled Level 2 condition and gender identification as fixed effects.

Gender identification did not moderate condition effects on EMA threat [*F* (1, 22.98) = 1.61, *p* = .217]. Gender identification did not significantly moderate the effect on resources [Condition × Gender Identification: *F* (1,23.01) = 0.29, *p* = .592] or demands [*F* (1, 22.94) = 2.62, *p* = .119].

Pilot Longitudinal Analyses

Main Analyses

Compared to the audiobook control, mindfulness participants reported lower psychological threat at posttest, which persisted at the 2-week follow-up [Figure S20; Condition × Assessment Interaction: *F* (2, 50) = 3.90, *p* = .027, *pseudo* R^2 = .27, 95% CI = [0.10, 0.54].

Figure S20. Long Term Effects of Mindfulness Training on Psychological Threat

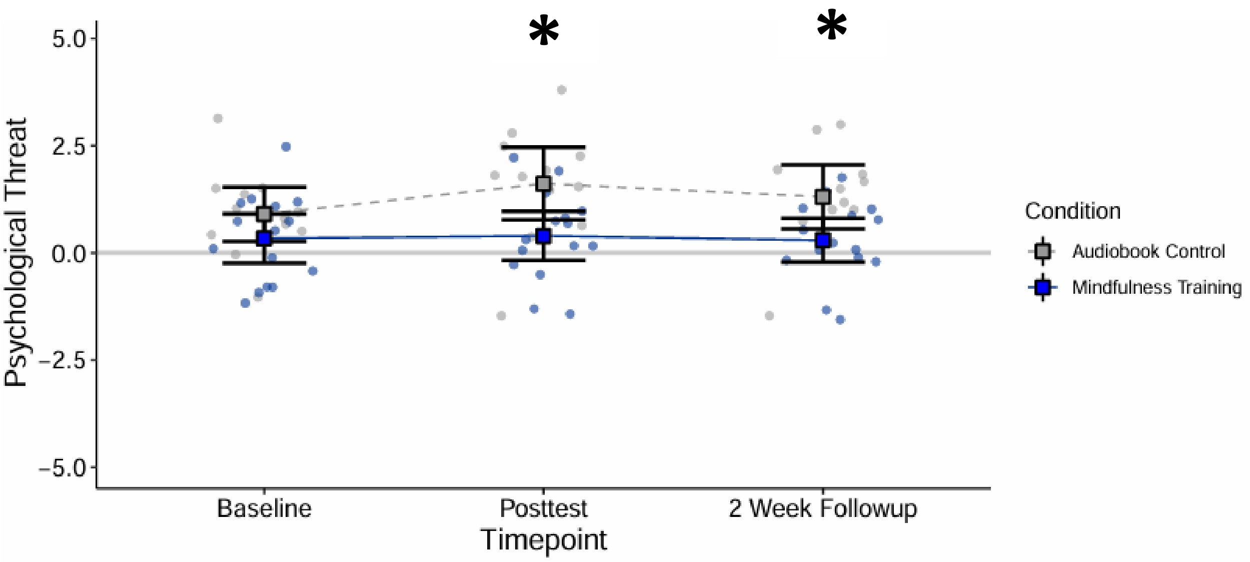

Note. Error Bars represent 95% CI. * *p* < .05; Graph presents raw values, not model adjusted values.

Analyses of resources and demands yielded results congruent with the EMA findings. Mindfulness participants reported greater coping resources than control participants across posttest and 2-week follow-up [Figure S21; Condition x Time Interaction: *F* (2,50) = 2.98, *p* = .059, *pseudo* R^2 = .26, 95% CI = [0.08, 0.53]. These findings are again consistent with a buffering effect whereby mindfulness mitigated longitudinal declines in coping resources.

Finally, conditions did not differ in demand appraisals [Figure S21; F (2, 50) = 1.68, p = .196, *pseudo* R^2 = .10, 95% CI = [0.03, 0.37].

Figure S21. Long Term Effects of Mindfulness Training on Resource & Demand Appraisals

A) Resources

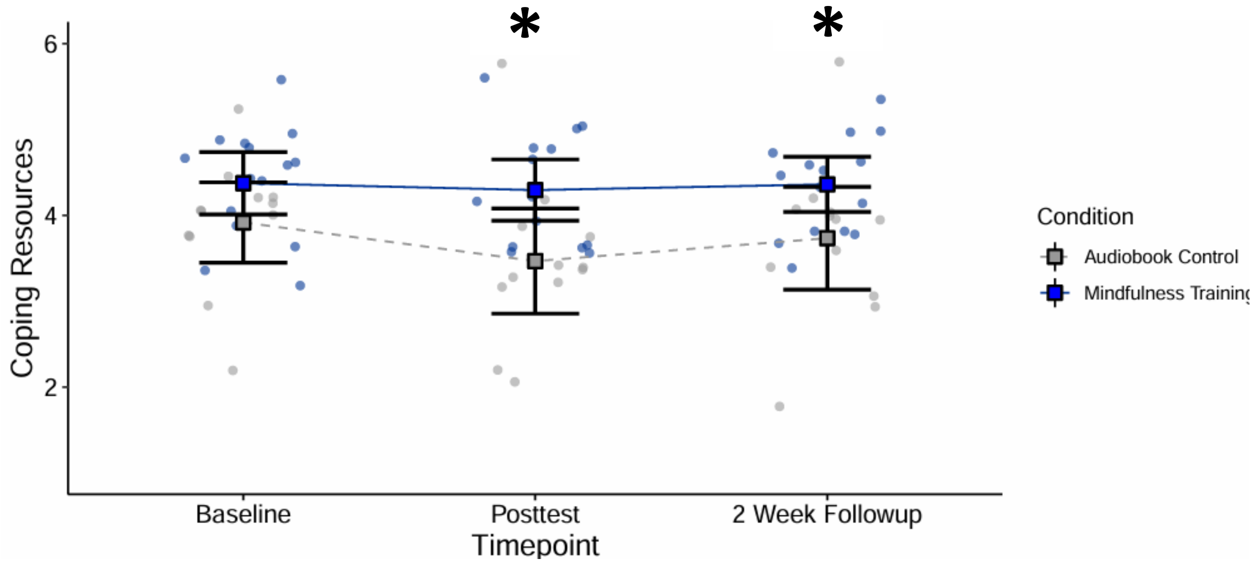

B) Demand Appraisals

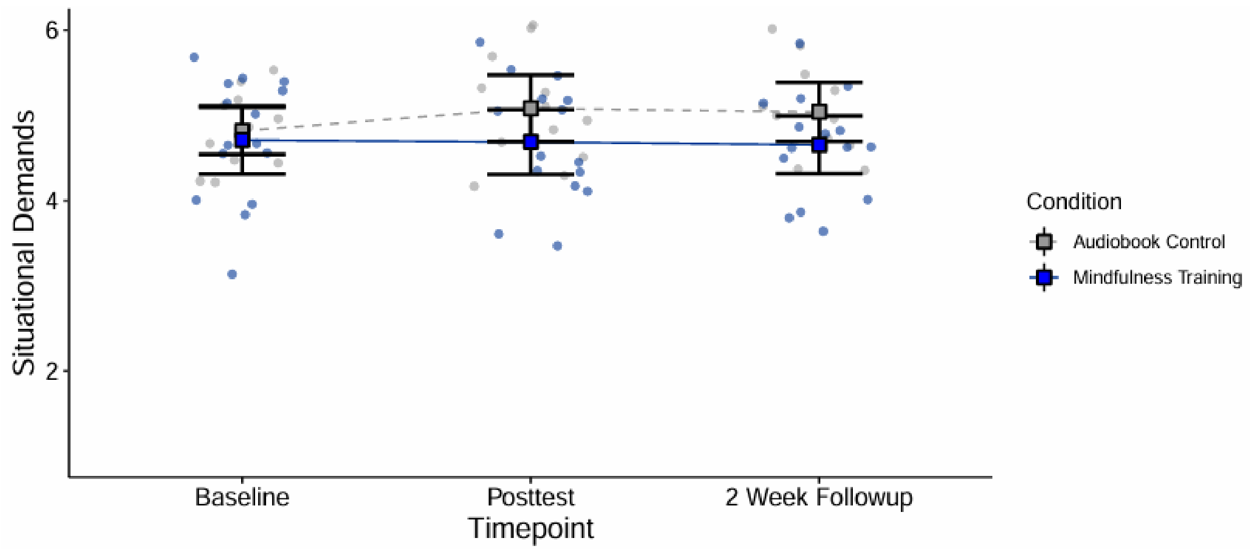

Note. Error Bars represent 95% CI. * $p < .05$; Graph presents raw values, not model adjusted values.

Table S21 provides details of the pairwise comparisons for threat and resources at each timepoint.

Table S21. Pairwise Contrasts for Longitudinal Resources

	**Threat**	**Resources**
Baseline	t (25) = 1.43, *p* = .165	t (25) = -1.71, *p* = .100
Posttest	*t* (25) = 2.68, *p* = .013	*t* (25) = -2.67, *p* = .013
2 Week Follow-Up	*t* (25) = 2.51, *p* = .019	*t* (25) = -2.12, *p* = .044

Longitudinal Robustness Analyses – without covariates

Mixed linear models, with maximum likelihood estimation, tested the longitudinal effects of condition (0 = audiobook control, 1 = mindfulness training) on threat, resources, or demands from baseline through posttest and follow-up surveys. Our model specified psychological threat as the Level 1 outcome and modeled Level 1 time as a categorical variable (baseline, posttest, and 2-week follow-up). As before, we modeled Level 1 intercept as a random effect, using variance components, and Level 2 condition as a fixed effect.

As predicted, students benefited from the mindfulness training relative to the audiobook control beyond the initial training week [Condition × Assessment Interaction: F (2, 50) = 3.90, p = .027, *pseudo* R^2 = .18]. Participants who received the mindfulness (vs. audiobook control) training reported lower psychological threat at posttest. This effect endured 2 weeks later. Notably, while students in the audiobook condition experienced increased psychological threat over this period, the mindfulness training buffered students from this increase.

Like what was found with the EMA data, mindfulness participants reported greater coping resources than control participants, and this effect was observed at posttest and 2-week follow-up assessment [Condition x Time Interaction: F (2,50) = 2.98, p = .060, *pseudo* R^2 = .18; *]*. Again, conditions did not differ in demand appraisals in both studies [F (2, 50) = 1.68, p = .196, *pseudo* R^2 = .07].

Longitudinal Moderation Analyses

Condition × Time × Analysis Classification

Mixed linear models, with maximum likelihood estimation, tested whether analysis classification (0 = systemically excluded groups, 1 = systemically advantaged group) moderated the longitudinal effects of condition (0 = audiobook control, 1 = mindfulness training) on threat from baseline through posttest and follow-up surveys. Our model specified psychological threat as the Level 1 outcome and modeled Level 1 time as a categorical variable (baseline, posttest, and 2-week follow-up). As before, we modeled Level 1 intercept as a random effect, using variance components, and Level 2 condition and analysis classification as fixed effects.

Analysis classification did not moderate the Condition × Assessment effect on psychological threat [3-Way Interaction: F (2,46) = 1.41, p = .255]. Analysis classification also did not moderate the effect on resources [3-Way Interaction: [F (2,46) = 1.56, p = .220] or demands [F (2, 46) = 0.27, p = .763].

Condition × Time × Racial Identification

Mixed linear models, with maximum likelihood estimation, tested whether racial identification (0 = students of color, 1 = white students) moderated the longitudinal effects of condition (0 = audiobook control, 1 = mindfulness training) on threat from baseline through posttest and follow-up. Our model specified psychological threat as the Level 1 outcome and modeled Level 1 time as a categorical variable (baseline, posttest, and 2-week follow-up). As before, we modeled Level 1 intercept as a random effect, using variance components, and Level 2 condition and racial identification as fixed effects.

Racial identification did not moderate the Condition × Assessment effect on psychological threat [3-Way Interaction: F (2,46) = 1.91, p = .313]. Racial identification also did not moderate the effect on resources [3-Way Interaction: [F (2,46) = 0.48, p = .621] or demands [F (2, 46) = 0.87, p = .422].

Condition × Time × Gender Identification

Mixed linear models, with maximum likelihood estimation, tested whether gender identification (0 = women and non-binary students, 1 = male students) moderated the longitudinal effects of condition

(0 = audiobook control, 1 = mindfulness training) on threat from baseline through posttest and follow-up surveys. Our model specified psychological threat as the Level 1 outcome and modeled Level 1 time as a categorical variable (baseline, posttest, and 2-week follow-up). As before, we modeled Level 1 intercept as a random effect, using variance components, and Level 2 condition and gender identification as fixed effects.

Gender identification did not moderate the Condition × Assessment effect on psychological threat [3-Way Interaction: F (2,46) = 0.54, p = .587]. Gender identification also did not moderate the effect on resources [3-Way Interaction: [F (2,46) = 1.24, p = .300] or demands [F (2, 46) = 0.00, p = .999].

References

1. A. Collins-Warfield, J. Niewoehner-Green, S. Scheer, K. Mills, Views of Struggling Students from Historically Excluded Groups on Academic Success and Instructor Support. *JPSS* **2**, 53–82 (2023).

2. N. Maghbouleh, A. Schachter, R. D. Flores, Middle Eastern and North African Americans may not be perceived, nor perceive themselves, to be White. *Proceedings of the National Academy of Sciences* **119**, e2117940119 (2022).

3. J. P. Jamieson, B. J. Peters, E. J. Greenwood, A. J. Altose, Reappraising Stress Arousal Improves Performance and Reduces Evaluation Anxiety in Classroom Exam Situations. *Social Psychological and Personality Science* **7**, 579–587 (2016).
